# Applicability of a miniaturized precipitation assay as an early-stage screening tool for prototype formulation development

**DOI:** 10.1016/j.ijpx.2026.100677

**Published:** 2026-10-01

**Authors:** Sara Mayer, Katharina Krollik, Christian Wagner, Andreas Lehmann, Jacob Spanheimer, Werner Weitschies

**Affiliations:** a Institute of Pharmacy, Department of Biopharmaceutics and Pharmaceutical Technology, University of Greifswald, Felix-Hausdorff-Straße 3, 17489 Greifswald, Germany; b Global Drug Product Development, Merck KGaA, Frankfurter Straße 250, 64293 Darmstadt, Germany

**Keywords:** Precipitation assay, In vitro-in vivo correlation, Intestinal absorption, Basic compounds, Prototype formulation development, Absorption predictions

## Abstract

Poor aqueous solubility is a frequently faced challenge in oral drug development. Therefore, the development of predictive in vitro tools for the selection of a suitable formulation is essential. For early formulation development, such an assay ideally requires minimal resources. To address this need, we evaluated the benefits of a previously published miniaturized two-compartment precipitation assay to assess formulation effects early in drug product development. The assay was originally developed for screening compounds for their absorption behavior during preclinical stage. Six poorly soluble weakly basic compounds (albendazole, ibrutinib, itraconazole, posaconazole, ritonavir, ziprasidone; two formulations each) and one poorly soluble neutral compound (fenofibrate; three formulations) were tested under simulated fasted conditions. Formulation differences found in vitro were compared to human pharmacokinetic data obtained from literature. From a qualitative perspective, the assay correctly reflected the in vivo observations for five out of the six basic compounds, as well as for fenofibrate. Quantitatively, the assay approximated the observed in vivo formulation effects for several compounds, including itraconazole, posaconazole, ritonavir, ziprasidone and fenofibrate, whereas the effect size was overestimated for albendazole and ibrutinib, likely reflecting potential assay limitations. These findings support the use of the miniaturized precipitation assay as a fit-for-purpose screening tool for early formulation development.

## Graphical abstract

**Figure:**
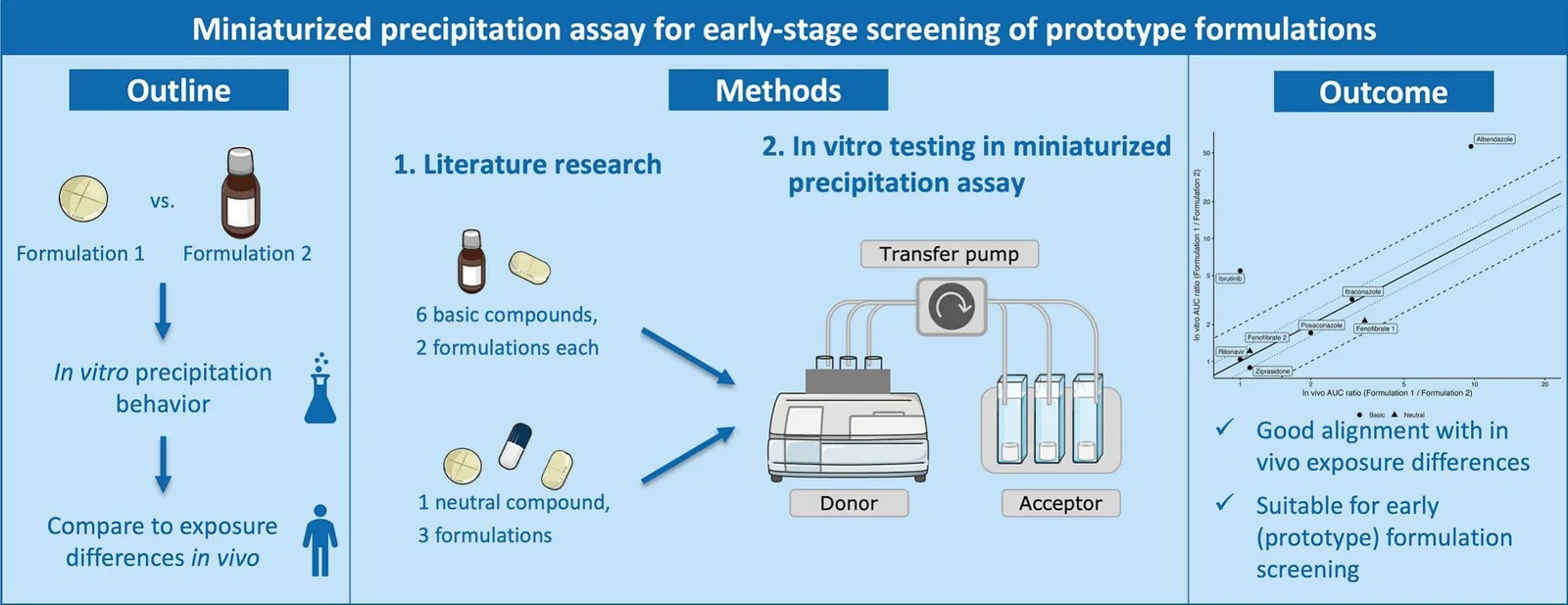

## 1 Introduction

Sufficient absorption in the gut is one of the key prerequisites for oral drug bioavailability and subsequently therapeutic efficacy. Poor solubility in gastrointestinal fluids and precipitation upon entry into the small intestine are considered important factors that may impair drug absorption (Augustijns et al., 2014; Brouwers et al., 2009; Kou et al., 2018). To address these challenges, various formulation strategies have been developed, including amorphous solid dispersions (ASDs), lipid-based formulations, and the formation of salts or co-crystals (Boyd et al., 2019). In this context, the early application of fit-for-purpose in vitro assays to characterize formulations in terms of their biopharmaceutical properties is essential for guiding formulation selection and development and accelerating timelines.

Especially for poorly soluble basic drugs or for supersaturating formulations such as ASDs, understanding precipitation behavior in the small intestine is particularly relevant for predicting oral absorption. Upon transfer from the acidic gastric environment to the near-neutral pH of the upper small intestine, drug supersaturation of weakly basic drugs can occur. If intestinal precipitation occurs from the supersaturated state, the rate and/or the extent of absorption may decrease (O'Dwyer et al., 2019). Recent work highlighted the importance of precipitation testing for weakly basic drugs early in drug discovery instead of relying on thermodynamic solubility measurements in Fasted State Simulated Intestinal Fluid (FaSSIF) only (Mayer et al., 2025a).

To date, various precipitation assays have been described, and development continues. One of the first transfer models was introduced by Kostewicz et al. and used donor and acceptor compartments connected by a peristaltic pump to simulate the transfer from gastric to intestinal conditions at physiologically relevant volumes (Kostewicz et al., 2004). These studies provided important mechanistic insight into pH-shift-induced precipitation and helped to establish two-stage precipitation testing as a relevant tool for poorly soluble, weakly basic drugs. Later assays often retained physiologically relevant volumes while incorporating increasingly complex, multi-compartment designs, such as the TNO Gastro-Intestinal Model (TIM), the biorelevant gastrointestinal transfer (BioGIT) model, and others (Andreas et al., 2018; Barker et al., 2014; Kourentas et al., 2016a). These assays use oral formulations such as capsules or tablets and are thus well suited for formulation development during the clinical stage, as they provide insights into luminal concentration profiles, assess potential food or proton pump inhibitor effects, and generate data for physiologically based biopharmaceutics modelling (PBBM). However, the resource requirements in terms of compound and formulation demand as well as setup complexity limit their applicability during preformulation stage. At this stage, i.e., where various formulation prototypes are screened to finally select the most promising first-in-human clinical formulation in terms of its biopharmaceutic performance, simpler and more resource-efficient tools are required. Some precipitation investigations have also been conducted in smaller two-stage test methods over the last years (Fiolka and Dressman, 2018; Jede et al., 2019a; O'Dwyer et al., 2019). However, to the best of our knowledge, many of them either only applied drug substances, used “dumping” approaches or organic solvents, or volumes that were still relatively large for very early development stages. Accordingly, there is a need for a miniaturized, two-stage precipitation assay that enables resource-efficient screening of formulation prototypes.

We previously developed a miniaturized precipitation assay specifically tailored to the drug discovery phase (Mayer et al., 2025b). The assay has demonstrated its usefulness as a screening tool for predicting oral drug absorption of basic drugs by integrating precipitation assay results alongside permeability measurements in our decision tree model, which classifies drug absorption into three categories (low, moderate, high) (Mayer et al., 2025a). However, the applicability of the miniaturized precipitation assay to drug products has not yet been evaluated.

Therefore, this study aimed to evaluate the previously developed miniaturized precipitation assay (Mayer et al., 2025b) as a screening tool for early formulation prototypes, particularly during the preformulation stage. For this purpose, formulations of the same compound were compared in vitro under simulated fasted conditions. Six poorly soluble basic compounds (albendazole, ibrutinib, itraconazole, posaconazole, ritonavir, ziprasidone; two formulations per drug) and one neutral compound (fenofibrate; three formulations) were included. The assay's ability to predict formulation effects was assessed by comparing differences in in vitro formulation performance with differences in systemic exposure observed in humans, particularly the area under the plasma concentration time curve (AUC). The key objective was not to establish a formal quantitative in vitro-in vivo correlation, but to assess whether the miniaturized assay can support early formulation ranking using limited material and reduced experimental complexity. Based on the outcome of this and of previous work (Mayer et al., 2025b; Mayer et al., 2025a), this study provides the formulation and biopharmaceutics scientist with a practical guidance on how to apply the miniaturized precipitation assay during discovery and preformulation stage.

## 2 Materials and methods

### 2.1 Materials

Itraconazole and posaconazole were purchased from Activate Scientific GmbH (Prien, Germany), ibrutinib from BLDpharm (Shanghai, China), ritonavir from abcr GmbH (Karlsruhe, Germany), ziprasidone hydrochloride monohydrate from AstaTech Inc. (Bristol, PA, USA), and fenofibrate from Cayman Chemical (Ann Arbor, MI, USA). Albendazole, sodium chloride, hydrochloric acid (1 M and 37%), 1 M sodium hydroxide, sodium hydroxide pellets, sodium dihydrogen phosphate monohydrate, acetonitrile, trifluoroacetic acid, acetic acid (100%), ammonia solution (25%), citric acid monohydrate, methylparaben, and propylene glycol were purchased from Merck KGaA (Darmstadt, Germany) or its subsidiaries. Hydroxypropyl-beta-cyclodextrin (HP-β-CD) (Kleptose® HPB Parenteral Grade) was purchased from Roquette (Lestrem, France), sorbitol solution (70%) and sodium saccharin from Caelo (Hilden, Germany), and 3F Powder from Biorelevant (London, United Kingdom). Marketed drug products were obtained from a public pharmacy and included Eskazole® tablets (albendazole, 400 mg, GSK plc), Imbruvica® tablets (ibrutinib, 140 mg, Janssen-Cilag GmbH), Sporanox® capsules (itraconazole, 100 mg, Janssen-Cilag GmbH), Noxafil® suspension (posaconazole, 40 mg/mL, Merck & Co.), Norvir® tablets (ritonavir, 100 mg, AbbVie Inc.) and Norvir® powder for suspension (ritonavir, 100 mg, AbbVie Inc.), and Zeldox® capsules (ziprasidone hydrochloride monohydrate, equivalent to 20 mg ziprasidone, Viatris) and Zeldox® suspension (ziprasidone hydrochloride monohydrate, equivalent to 10 mg/mL ziprasidone, Viatris).

### 2.2 Selection of compounds

A key aspect of this study was the selection of suitable model compounds and formulations for validation of the miniaturized precipitation assay for predicting formulation effects. To focus on intestinal precipitation risk, poorly soluble basic compounds were prioritized. To allow for formulation comparison, one key requirement was the availability of human pharmacokinetic data for at least two formulations. Within the constraints imposed by data availability, we aimed to include formulation pairs spanning a range of reported in vivo outcomes, from minimal to pronounced formulation effects. Literature searches were conducted using PubMed, Google Scholar, and the Clinical Pharmacology and Biopharmaceutics Reviews available on the U.S. Food and Drug Administration (FDA) website (U.S. Food and Drug Administration, n.d.). Relevant search terms included: weakly basic drug, pH-dependent solubility, supersaturation, precipitation, crossover study, mass balance study, relative bioavailability, fasted state, enabling formulation, and formulation comparison.

### 2.3 Analytical methods

Dissolved drug concentrations were quantified by Ultra High Performance Liquid Chromatography (UHPLC) using a Waters ACQUITY UPLC H-Class PLUS system (Waters Corporation, Milford, MA, USA) equipped with a BEH C18 column (2.1 × 50 mm, 1.7 μm, 130 Å, Waters Corporation). A generic gradient method with a gradient changing from 95% mobile phase A (water with 0.1% trifluoroacetic acid) to 95% mobile phase B (acetonitrile) within 2.5 min, followed by a return to initial conditions at 3 min was applied for most compounds. The total run time was 4 min. Adaptations to this method were made for ritonavir and ziprasidone (see supplementary materials for additional information). Details about the determination of the limits of quantification (LOQ) are provided in the supplementary materials, and the measured LOQ values as well as the detection wavelengths are listed in Table S1.

### 2.4 Preparation of media

Simulated Gastric Fluid sine pepsin pH 2.0 (SGFsp pH 2.0) was prepared by dissolving sodium chloride in ultrapure water to a final concentration of 2 g/L and adjusting to pH 2.0 with 1 M hydrochloric acid. Double-buffered, double-concentrated Fasted State Simulated Intestinal Fluid (FaSSIFdbdc) was prepared based on the FaSSIF-V1 composition reported by Klein and originally described by Galia et al. (Galia et al., 1998; Klein, 2010), but with certain adaptations. To compensate for the dilution with SGFsp pH 2.0 during the transfer, the concentrations of sodium hydroxide, sodium dihydrogen phosphate monohydrate, and the 3F powder were doubled, while the amount of sodium chloride in the buffer was kept constant. The buffer concentrations were: 0.84 g/L sodium hydroxide, 7.90 g/L sodium dihydrogen phosphate monohydrate, and 6.186 g/L sodium chloride. The pH was adjusted to 6.5 using 1 M hydrochloric acid or 1 M sodium hydroxide. 3F Powder was dissolved in this buffer to a total concentration of 4.48 mg/mL. Where required, pH or buffer concentration was modified (see 2.6.1 Compound-specific adaptations).

### 2.5 Preparation of formulations

Where no commercial formulation was available, test formulations were prepared in-house to reflect the respective clinical formulation as closely as possible. For albendazole, the formulation was prepared according to Rigter et al. (Rigter et al., 2004). For a final volume of 10 mL, 30 mg albendazole, 4 g HP-β-CD, 105 mg citric acid monohydrate, and 15 mg methyl parahydroxybenzoate were used. As the formulation was found to be a suspension rather than a solution, several preparation techniques were evaluated, including different orders of component mixing, sonication in an ultrasonic bath, and heating. In the final procedure, all components except albendazole were dissolved in a portion of water, after which albendazole was added. The formulation was then brought to a final volume of 10 mL with water and stirred overnight. For use in the precipitation assay, the albendazole formulation was treated as a solution. For ibrutinib, the solution formulation was prepared as a 5 mg/mL solution in 30% HP-β-CD, as described in a mass balance study (Scheers et al., 2015). Because the commercial itraconazole oral solution (Sporanox®) was discontinued, the test formulation was prepared according to the Sporanox® patent, but without adding the flavors (Janssen Pharmaceutica N.V. 2340 Beerse, 1995). For posaconazole, administration conditions reported by Hens et al. (Hens et al., 2016) were reproduced. For the neutral condition, 1 mL of Noxafil® suspension was dispersed in 240 mL of tap water adjusted to pH 7.1 with 1 M sodium hydroxide (NaOH); for the acidic condition, tap water was adjusted to pH 1.6 with 1 M hydrochloric acid (HCl).

### 2.6 Precipitation assay

The precipitation assay used herein (Mayer et al., 2025b) applies 1.2 mL each for the donor and the acceptor compartment. Briefly, the donor compartment contained the formulation dissolved (ibrutinib solution, itraconazole solution) or dispersed (all remaining formulations) in SGFsp pH 2.0. An aliquot of 1.2 mL was transferred into a 10 mm cuvette containing 1.2 mL FaSSIFdbdc as acceptor medium. Transfer from the donor to the acceptor was performed with peristaltic pumps (Ismatec Reglo ICC with Ismatec Tygon LMT 55 tubes) using zero-order kinetics with a transfer time of 25 min. The cuvettes were maintained at 37 °C, and the test media were stirred at 500 rpm in an ultraviolet-visible (UV–Vis) spectrophotometer (Cary 3500, Agilent Technologies, Santa Clara, USA). All experiments were conducted in triplicate. For standardization purposes, a gastric volume of 250 mL was assumed, approximating the residual gastric volume plus one glass of water (Mudie et al., 2014). This approach allowed for standardization of the test protocol and accounted for unknown drinking volumes and interindividual variability in gastric residual volume. For solid dosage forms such as tablets or capsules, the entire dose was subjected to gastric dissolution for 15 min in 250 mL SGFsp pH 2.0 using a United States Pharmacopeia type II (USP II) dissolution apparatus (DT 726, Erweka GmbH, Langen, Germany) with mini vessels stirred by mini paddles at 75 rpm and 37 °C. After 15 min, an aliquot of approximately 10 mL was transferred to a Thermomixer Comfort (Eppendorf SE, Hamburg, Germany), which served as the donor compartment of the miniaturized setup (Mayer et al., 2025b). The donor compartment was maintained at 37 °C and shaken at 500 rpm, and pumping was started immediately. For liquid formulations, the administered dose volume was assumed to be diluted with SGFsp pH 2.0 to a total gastric volume of 250 mL in vivo. For the experiments, the ratio of formulation to medium volume was proportionally downscaled to a total volume of 10 mL. Suspension formulations were preincubated in a Thermomixer Comfort for 15 min at 37 °C and 500 rpm, after which pumping was initiated. In contrast, solution formulations were stirred at room temperature. As the drugs from these formulations were already fully dissolved prior to the transfer and no temperature-dependent dissolution step in the donor compartment was required, handling of solution formulations was adopted from the previously established miniaturized precipitation assay (Mayer et al., 2025b). The initial dissolution step in 250 mL SGFsp pH 2.0 was applied specifically to the marketed solid dosage forms included in the present evaluation study to enable comparison with published human pharmacokinetic (PK) data. In a preformulation setting, early prototype formulations can be tested directly at the miniaturized scale using reduced amounts of formulation material.

For quantification of dissolved drug, manual sampling followed by UHPLC analysis was used. This adaptation was necessary because the low solubility of several compounds precluded the development of a suitable UV method, some formulations reached concentrations outside a suitable UV range, and excipients interfered with optical detection. Samples were collected at 2.5, 5, 7.5, 10, 15, 20, 25, 30, 45, and 60 min and centrifuged at 15,000 rpm for 2 min at 37 °C. For the ibrutinib solution, samples were collected only at 5, 15, 25, 45, and 60 min and centrifuged under the same conditions. Subsequently, the supernatant was diluted with a suitable acetonitrile/water extraction mixture prior to UHPLC analysis (“2.3 Analytical methods”). An additional sample was taken either from the donor compartments or the dissolution vessels to determine dissolved concentrations in SGFsp pH 2.0.

To compare formulations, relative area under the curve (AUC_rel_) values over 45 min were calculated as described by Mayer et al. (Mayer et al., 2025b). Two reference metrics were used: AUC_rel_dose_, where the theoretical profile is based on the assumption that the entire dose is dissolved and transferred (100% = total dose), and AUC_rel_dissolved_, where the theoretical profile is scaled to the amount dissolved in the donor. Thus, AUC_rel_dose_ reflects deviations from complete dose dissolution (including gastric limitations), whereas AUC_rel_dissolved_ quantifies changes occurring after transfer in the acceptor relative to the dissolved input. Theoretical acceptor concentrations were calculated from donor concentrations and pump transfer rate while accounting for changes in drug amount and volume due to sampling. AUC_rel_ was then obtained as the ratio of measured to theoretical AUC.

#### 2.6.1 Compound-specific adaptations

For albendazole, assay conditions were adapted to reflect the large administration volume used clinically. A gastric volume of 442 mL and an intestinal volume of 250 mL were assumed (Rigter et al., 2004), and assay volumes were scaled accordingly to a total of 2.4 mL across both compartments. Because of the larger acidic input, the pH of the phosphate buffer for the acceptor medium was increased to 7.0 using 1 M NaOH. Since the citric acid in the solution formulation added substantial buffering capacity, two additional conditions were tested: (1) A five-fold increase in buffer concentration and (2) replacement of citric acid with hydrochloric acid. For the albendazole tablet, the stirring rate was increased from 75 rpm to 220 rpm for 1 min prior to sample withdrawal. For itraconazole, capsule contents were removed from the shells, and the required amount was weighed directly into the donor compartment, followed by addition of preheated SGFsp pH 2.0. This approach was used because it has been reported that removal of the capsule shell does not affect the dissolution behavior (Rudolph et al., 2025).

For posaconazole, the acceptor medium was again adjusted to pH 7.0 but only for the acidic condition. For ritonavir, tablet disintegration was incomplete under standard conditions; therefore, an additional experiment was performed using ground tablets to simulate complete tablet disintegration. Tablets were ground using a mortar and pestle. For the ground tablet and powder for suspension, the required amount of powder was weighed directly into the donor compartment, followed by addition of preheated SGFsp pH 2.0. In case of the intact ritonavir tablet, the stirring rate was increased from 75 rpm to 220 rpm for 1 min prior to sample withdrawal to improve sample homogeneity.

### 2.7 Statistical evaluation

For each of the six basic compounds, absolute AUC values (45 min) were compared between the two formulations using an unpaired two-tailed Welch's *t*-test. Welch's *t*-test was selected because it does not assume equal variances between groups and is therefore more suitable for small sample sizes, where variance equality cannot be reliably tested (Gastwirth et al., 2009; Ruxton, 2006; Welch, 1947). Normal distribution of the data was assumed. To control the false discovery rate across the six comparisons, *p*-values were adjusted using the Benjamini-Hochberg (BH) procedure at α = 0.05 (Benjamini and Hochberg, 1995). For fenofibrate, three formulations were tested; however, the analysis was limited to the two predefined comparisons aligned with the in vivo reference (145 mg vs. 200 mg; 160 mg vs. 200 mg). These comparisons were also evaluated using unpaired two-tailed Welch's *t*-tests, with BH adjustments applied across the two tests (α = 0.05). Since the formulations were predefined and compound-specific, the statistical analysis focused on predefined pairwise comparisons within each compound.

## 3 Results

Six poorly soluble weakly basic compounds with two formulations per drug were selected. Published human data indicated little or no relevant formulation effect for three compounds (ibrutinib, ritonavir, and ziprasidone), whereas the other three showed exposure differences ranging from 2.0-fold to 9.7-fold (albendazole, itraconazole, and posaconazole). In addition, one neutral compound (fenofibrate) with three formulations was included to extend the evaluation to a neutral poorly soluble compound. Key physicochemical properties of these compounds are provided in Table S2 in the supplementary materials. Concentration-time profiles and summary metrics from the miniaturized precipitation assay, as well as the in vivo reference data, are shown in Table 1, Fig. 1, Fig. 2, and Fig. 3. Absolute area under the curve (AUC_abs_) values from the precipitation assay are listed in Table S3 in the supplementary materials.

**Table 1:** Investigated formulations with in vivo and in vitro results.

Compound	Dose (mg)	Formulation	In vitro AUC_rel_dose_ (%)	In vitro AUC_rel_dissolved_ (%)	In vitro ratio (F1/F2) based on AUC_abs_	*p*-value Welch's test based on AUC_abs_ (after BH-correction)	In vivo ratio (F1/F2)
Albendazole	800	HP-β-CD solution (F1)	81.9 ± 1.3	95.4 ± 1.5	56.3	<0.05	9.7 (AUC_0-∞_) (Rigter et al., 2004)
		Eskazole® tablet (F2)	1.45 ± 0.22	30 ± 5			
Ibrutinib	140	HP-β-CD solution (F1)	101.8 ± 1.8	n/a	5.5	<0.05	No difference (See “Ibrutinib” in Section 3 for details)
		Imbruvica® tablet (F2)	17.8 ± 0.5	59.3 ± 1.7			
Itraconazole	200	Sporanox® HP-β-CD solution (F1)	10.4 ± 2.9	n/a	3.2	0.055	3.0 (AUC_0-8h_) (Brouwers et al., 2017)
		Sporanox® capsule (ASD) (F2)	3.27 ± 0.28	99 ± 9			
Posaconazole	40	Noxafil® suspension (acidified) (F1)	6.5 ± 0.5	8.9 ± 0.7	1.7	<0.05	2.0 (AUC_0-8h_) (Hens et al., 2016)
		Noxafil® suspension (neutral) (F2)	3.79 ± 0.05	139.0 ± 1.9			
Ritonavir	100	Norvir® powder for suspension (ASD) (F1)	30.1 ± 0.8	84.5 ± 2.0	1.0	0.11 (ground)	No difference (Xu et al., 2017)
		Norvir® tablet (ASD) (F2)	28.95 ± 0.14 (ground)	132.9 ± 0.7 (ground)			
Ziprasidone (hydrochloride monohydrate)	20 (free base)/ 22.64 (salt)	Zeldox® capsule (F1)	15.5 ± 0.7	170 ± 7	0.9	<0.05	1.1 (AUC_0-∞_) (FDA, Center for Drug Evaluation and Research, 2005)
		Zeldox® suspension (F2)	17.4 ± 0.8	232 ± 11			
Fenofibrate	145	Lipidil 145 one® tablet (nanosized)	9.0 ± 0.5	128 ± 6	2.1 (145 mg vs. 200 mg, dose-corrected) 1.2 (160 mg vs. 200 mg)	<0.05 (145 mg vs. 200 mg) 0.12 (160 mg vs. 200 mg) (Fenofibrate results were treated separately from the basic compounds.)	3.4 (145 mg vs. 200 mg, dose-corrected AUC_0-8h_) (Hens et al., 2015) 1.1 (160 mg vs. 200 mg, AUC_0-∞_) (FDA, Center for Drug Evaluation and Research, 2001)
	160	Lipidil-Ter® tablet (micronized and microcoated)	6.4 ± 0.5	46 *10 ± 4 *10			
	200	Lipidil® capsule (micronized)	4.2 ± 0.7	74 *10 ± 12 *10			

All in vitro AUC values refer to a 45 min time interval and are given as mean ± SD, *n* = 3. For albendazole solution, the results from the initial setup are displayed. For calculation of the in vitro ratio of ritonavir, the ground tablet was used. F1 = Formulation 1, F2 = Formulation 2, HP-β-CD = Hydroxyprobyl-beta-cyclodextrin, ASD = amorphous solid dispersion, AUC = area under the curve, AUC_abs_ / AUC_rel_dose_ / AUC_rel_dissolved_ = absolute AUC/ relative AUC based on dose / relative AUC based on the dissolved amount in the donor, BH = Benjamini-Hochberg, n/a = not applicable, SD = standard deviation.

**Fig. 1:**
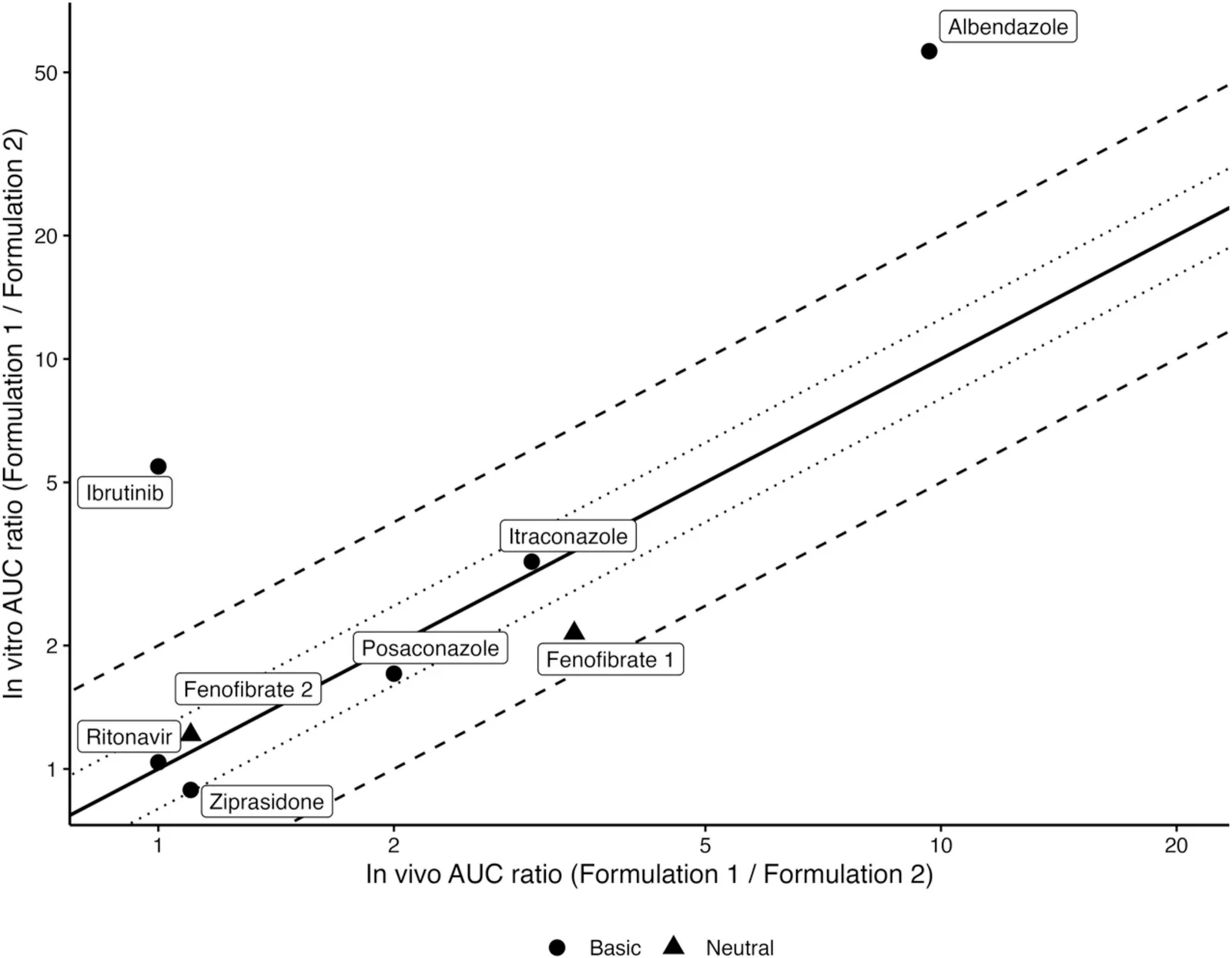
In vitro-in vivo comparison of AUC ratios for six basic compounds (●) and one neutral compound (▲). The solid line represents the line of identity, indicating perfect agreement between in vivo and in vitro ratios. Dotted and dashed lines indicate a 1.25-fold and 2-fold deviation from the line of identity. Both axes are shown on a logarithmic scale. Fenofibrate 1 represents the comparison of the 145 mg (Lipidil 145 one® tablet) and 200 mg (Lipidil® capsule) formulations; Fenofibrate 2 represents the comparison of the 160 mg (Lipidil-Ter® tablet) and 200 mg (Lipidil® capsule) formulations. Ziprasidone was tested as ziprasidone hydrochloride monohydrate. For further details on the tested formulations, refer to Table 1. AUC = area under the curve.

**Fig. 2:**
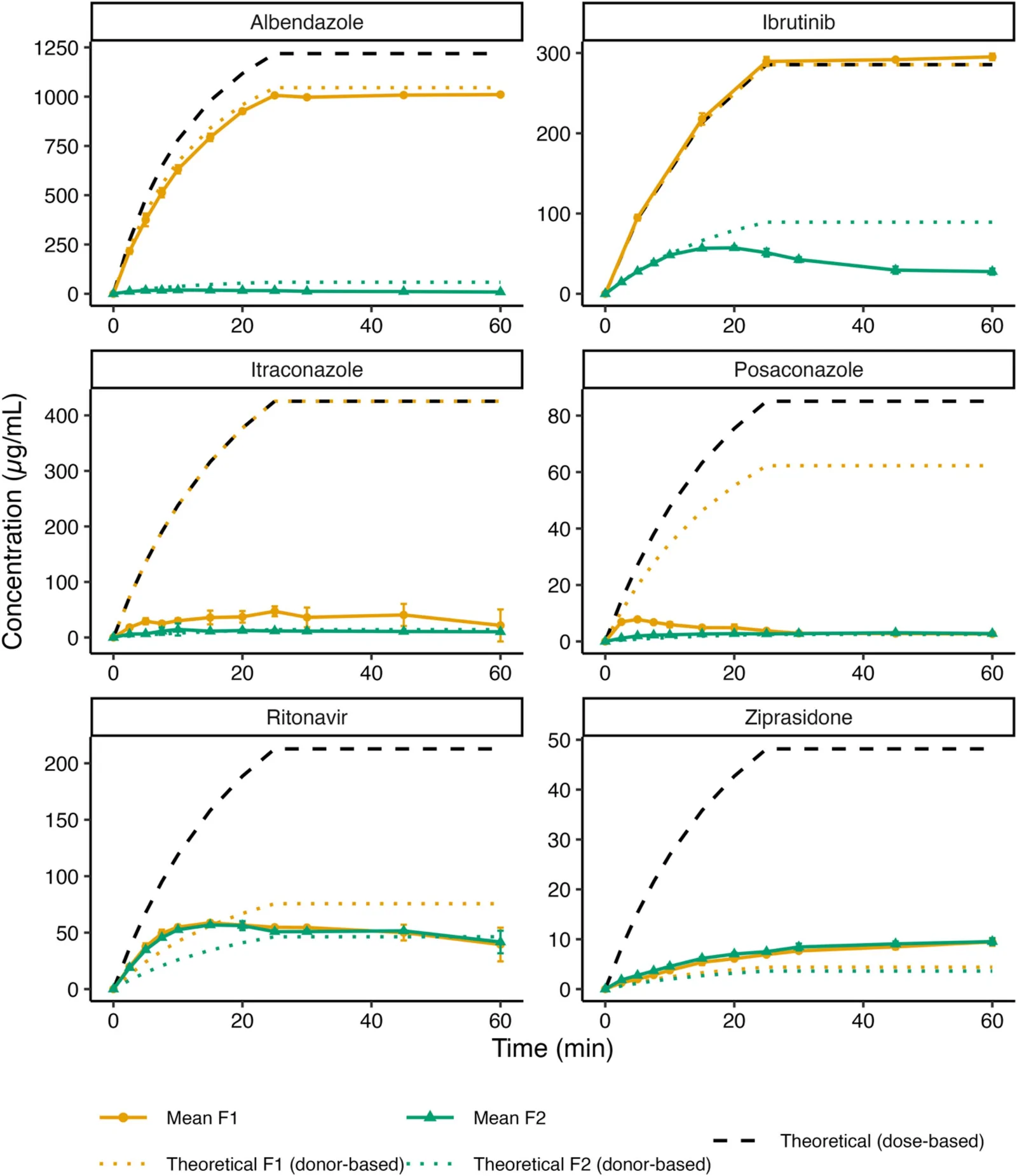
Concentration profiles of the six basic compounds with two formulations each, given as mean ± SD, *n* = 3. Dashed lines: dose-based theoretical profiles, assuming complete dissolution in the donor compartment and no precipitation during or after transfer. Dotted lines: donor-dissolved-based theoretical profiles, calculated from the amount dissolved in SGFsp pH 2.0 and assuming no further dissolution or precipitation during or after transfer. Details of F1 and F2 are given in Table 1. For the albendazole solution, results from the standard setup are displayed, and for ritonavir, results from the ground tablet are shown. Ziprasidone was tested as ziprasidone hydrochloride monohydrate. SD = standard deviation, SGFsp = Simulated Gastric Fluid sine pepsin, F1 = Formulation 1, F2 = Formulation 2.

**Fig. 3:**
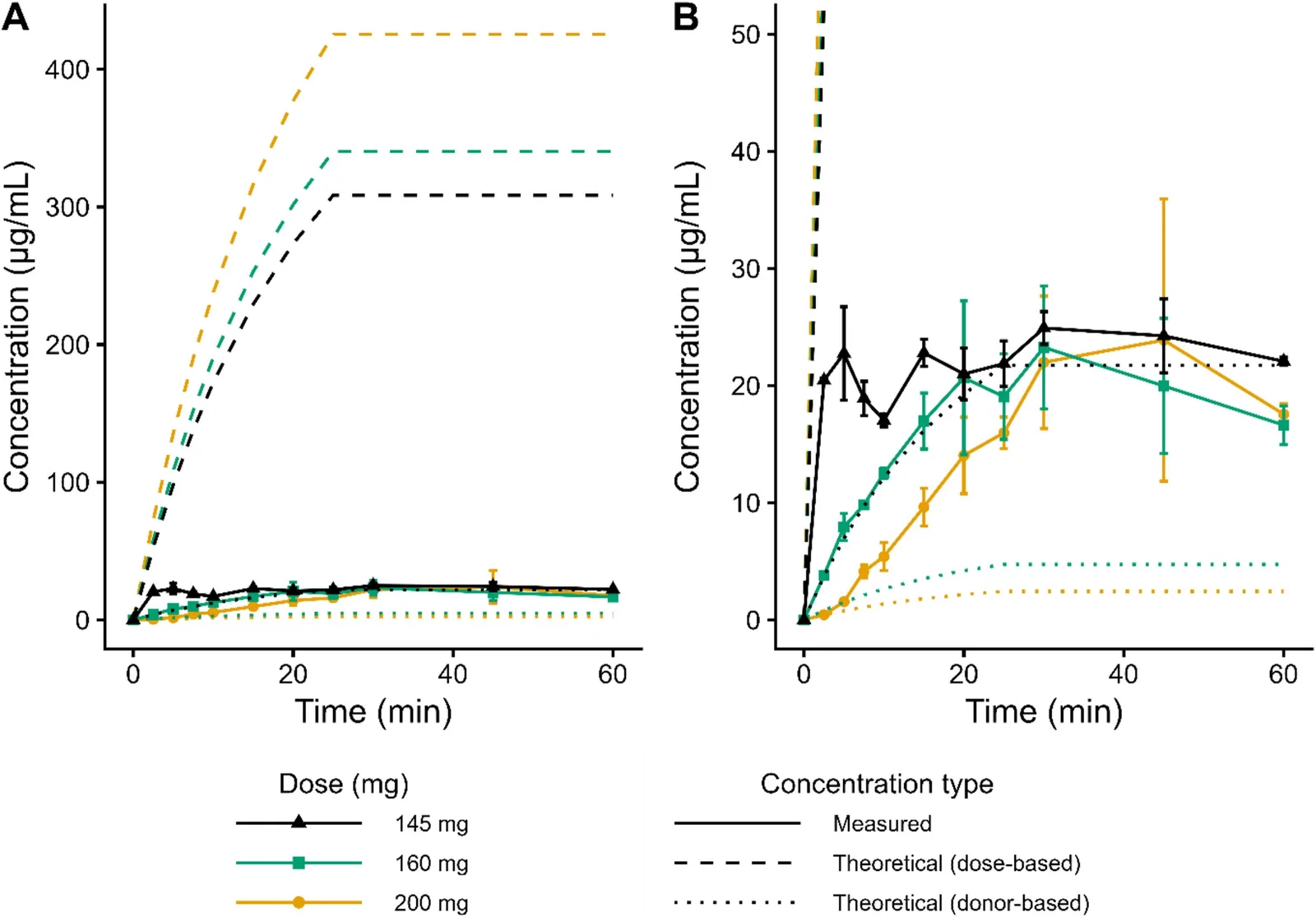
Concentration profiles of fenofibrate formulations, given as mean ± SD, *n* = 3. A: Full scale, B: Zoomed-in view. The tested formulations were the capsule with micronized fenofibrate (Lipidil®, 200 mg), the tablet with microcoated fenofibrate (Lipidil-Ter®, 160 mg), and the tablet with nanosized fenofibrate (Lipidil 145 one®, 145 mg). Dashed lines indicate dose-based theoretical profiles, assuming complete dissolution in the donor compartment and no precipitation during or after transfer. Dotted lines indicate donor-dissolved-based theoretical profiles, calculated from the amount dissolved in SGFsp pH 2.0 and assuming no further dissolution or precipitation during or after transfer. SD = standard deviation, SGFsp = Simulated Gastric Fluid sine pepsin.

### 3.1 Overall comparison

Formulation differences observed in vitro were broadly in line with the in vivo findings for itraconazole, posaconazole, ritonavir, ziprasidone, and fenofibrate (Fig. 1, Table 1). For albendazole, the same formulation was found to be superior in both in vitro and in vivo comparisons; however, the magnitude of the difference was considerably greater in vitro. In contrast, for ibrutinib, the assay indicated a formulation difference in vitro that was not apparent from the available human PK data.

#### 3.1.1 Albendazole

In a crossover study at a dose level of 800 mg, a HP-β-CD solution of albendazole yielded a 9.7-fold higher relative bioavailability (AUC_0-∞_) than the marketed tablet (Eskazole®) (Rigter et al., 2004). Using the standard conditions in the precipitation assay, the AUC ratio was 56.3. The difference in AUC_absolute_ was statistically significant (Table 1, Fig. 2). Notably, the cyclodextrin formulation resulted in a suspension, with approximately 70–80% of the dose dissolved. Nevertheless, this formulation resulted in higher acceptor concentrations than the tablet, and no precipitation was observed. Accordingly, AUC_rel_dissolved_ was 95.4%, while AUC_rel_dose_ was 81.9% using the standard conditions (Table 1, Fig. 2). Due to the buffering effect of the formulation, the acceptor pH was 3.4 after transfer. Therefore, two additional assay conditions were evaluated. Increasing the buffer concentration of the acceptor medium resulted in a final pH of approximately 5.9, while replacing citric acid with hydrochloric acid resulted in a final pH of approximately 6.6. Under both control conditions, comparable concentration–time profiles were observed (see supplementary materials Table S3 and Fig. S1). For the tablet, about 5% of the dose was dissolved in SGFsp pH 2.0 after 15 min. Precipitation in the acceptor compartment was observed after approximately 5 min. Acceptor concentrations reached 19 μg/mL and decreased to 9 μg/mL at 60 min, corresponding to an AUC_rel_dissolved_ of 30% and an AUC_rel_dose_ of 1.45%.

#### 3.1.2 Ibrutinib

For ibrutinib, a HP-β-CD solution was compared to the marketed tablet formulation (Imbruvica®) at a dose level of 140 mg. Available clinical data indicate no relevant difference in the relative bioavailability between the two formulations, and complete absorption can be assumed for both formulations. In a mass balance study (Scheers et al., 2015), the plasma concentration of the tablet was marginally higher than that of the solution (FDA, Center for Drug Evaluation and Research, 2018). In contrast, the miniaturized precipitation assay showed a 5.5-fold difference between the formulations. The solution achieved higher acceptor concentrations than the tablet and yielded a statistically significantly different AUC_absolute_ (Table 1, Fig. 2). The solution remained fully dissolved throughout the transfer with an AUC_rel_dose_ close to 100%. In contrast, the tablet showed incomplete dissolution in SGFsp pH 2.0, with about 30% of the dose dissolved before transfer. Precipitation in the acceptor compartment was observed after approximately 10 min. Acceptor concentrations reached 57 μg/mL and declined to 28 μg/mL at 60 min. This corresponded to an AUC_rel_dose_ of 17.8% and an AUC_rel_dissolved_ of 59.3%.

#### 3.1.3 Itraconazole

For itraconazole, a HP-β-CD solution was compared to the marketed capsule formulation (Sporanox®) at a dose level of 200 mg. In a crossover study, the commercial oral solution (Sporanox®) showed a 3.0-fold higher AUC_0-8h_ than the capsule (Brouwers et al., 2017). In the precipitation assay, the corresponding in vitro difference was 3.2-fold. Acceptor concentrations were low for both formulations, with AUC_rel_dose_ values below 15% in both cases (Table 1, Fig. 2). Nevertheless, the solution consistently reached higher concentrations compared to the capsule. The AUC_absolute_ difference between formulations was statistically not significant. For the solution, precipitation was observed immediately after the start of transfer. For the capsule, approximately 3% of the dose was dissolved in SGFsp pH 2.0 before transfer, and this fraction remained in solution throughout the experiment, corresponding to an AUC_rel_dissolved_ close to 100%.

#### 3.1.4 Posaconazole

For posaconazole, an acidified version of the marketed suspension (Noxafil®) was compared to a neutral version of the same suspension at a dose level of 40 mg. In a crossover study, the acidified suspension resulted in a 2.0-fold higher plasma AUC_0-8h_ than the neutral suspension (Hens et al., 2016). In vitro, the corresponding difference was 1.7-fold. Overall acceptor concentrations were low for both formulations, with AUC_rel_dose_ values below 10% (Table 1, Fig. 2). The acidified suspension achieved higher maximum concentrations and a higher AUC_rel_dose_ than the neutral suspension. The AUC_absolute_ difference between the two formulations was statistically significant. Differences were also observed in the dissolved fraction prior to transfer. For the acidified suspension, approximately 70% of the dose was dissolved before and after adding SGFsp pH 2.0, whereas the neutral suspension contained about 3% dissolved drug in the donor before transfer. During transfer of the acidified suspension, precipitation was observed immediately. For the neutral suspension, prolonged drug dissolution during the transfer phase led to acceptor concentrations above the theoretical profile (donor-based), leading to an AUC_rel_dissolved_ of 139%. At 30 min after the start of transfer, concentrations were below 3 μg/mL and comparable for both formulations.

#### 3.1.5 Ritonavir

For ritonavir, the marketed tablet (Norvir®) was compared to the marketed powder for oral suspension (Norvir®) at a dose level of 100 mg. These formulations have been reported to be bioequivalent under fasted conditions (Xu et al., 2017). Because tablet disintegration in SGFsp pH 2.0 was incomplete, both intact and ground tablets were tested. AUC_rel_dose_ values were in a comparable range for all three conditions, with 30.1% for the powder for suspension, 28.95% for the ground tablet, and 23.3% for the intact tablet (Table 1, Fig. 2). With about 36% of the dose dissolved, the powder for suspension showed the highest dissolved fraction in SGFsp pH 2.0 before the transfer, whereas 22–26% of the dose was dissolved when the intact and ground tablets were tested. Although the dissolved fraction in the donor was lower for the ground tablet than for the powder for suspension, both formulations showed similar acceptor concentrations. The intact tablet showed lower initial concentrations after start of the transfer than both the powder for suspension and the ground tablet and followed the theoretical profile; minor precipitation was observed after 25 min, corresponding to an AUC_rel_dissolved_ of 90% (see supplementary materials Table S3 and Fig. S2). 15 min after start of transfer, mean acceptor concentrations ranged from 40 to 59 μg/mL across all formulations. Statistical analysis showed a significant difference between the powder and the intact tablet, but not between the powder and the ground tablet.

#### 3.1.6 Ziprasidone

For ziprasidone, the marketed capsule and suspension (Zeldox®) were compared at a dose level of 20 mg. In a crossover study, the capsule yielded a 1.1-fold higher plasma AUC than the suspension (FDA, Center for Drug Evaluation and Research, 2005). In the precipitation assay, the corresponding in vitro ratio was 0.9. Acceptor concentrations were low and comparable for both formulations, with a statistically significant difference between formulations (Table 1, Fig. 2). No precipitation was detected for either formulation. Instead, acceptor concentrations continued to increase during the experiment, resulting in AUC_rel_dissolved_ values above 100% (Table 1). For both formulations, the dissolved fraction in the donor compartment before transfer was below 10% of the dose. For the suspension, the dissolved amount of compound in the donor and the 25 min value were based on two measurements only, as the third values appeared implausibly high and exceeded the maximum achievable concentration.

#### 3.1.7 Fenofibrate

For fenofibrate, three marketed formulations were compared: Lipidil® 200, a capsule containing 200 mg micronized fenofibrate; Lipidil-Ter® 160, a tablet containing 160 mg microcoated fenofibrate; and Lipidil 145 one®, a tablet containing 145 mg nanosized fenofibrate. In a crossover study, the 160 mg tablet yielded a 1.1-fold higher plasma AUC than the 200 mg capsule (FDA, Center for Drug Evaluation and Research, 2001). In another crossover study, the 145 mg tablet yielded an almost 3.4-fold higher dose-corrected plasma AUC than the 200 mg capsule. In vitro, the corresponding differences were 1.2-fold for the 160 mg tablet versus the 200 mg capsule (based on absolute AUC) and 2.1-fold for the 145 mg tablet versus the 200 mg capsule (based on relative AUC). Acceptor concentrations were low for all three formulations, with AUC_rel_dose_ values below 10% (Table 1, Fig. 3). The 145 mg tablet containing nanosized fenofibrate showed the highest AUC_rel_dose_ (9.0%), followed by the 160 mg tablet containing microcoated fenofibrate (6.4%) and the 200 mg capsule containing micronized fenofibrate (4.2%). Dissolution in SGFsp pH 2.0 after 15 min was incomplete for all formulations, with approximately 7% of the dose dissolved for the 145 mg tablet, less than 1.5% for the 160 mg tablet, and less than 1% for the 200 mg capsule. During transfer, dissolution of all three formulations continued in the acceptor compartment, resulting in AUC_rel_dissolved_ values above 100% (Table 1). Although measured concentrations for the 200 mg capsule at 2.5 min were below the LOQ, signal-to-noise ratio assessment suggested that quantification might still be feasible and these values were therefore retained for further analysis.

## 4 Discussion

This study assessed whether a miniaturized precipitation assay can support the ranking of prototype formulations during early development by capturing formulation-dependent absorption differences. Across the evaluated dataset, the assay proved to be useful as a qualitative screening tool for formulation comparison and, in several cases, also approximated the magnitude of the formulation effect observed in vivo reasonably well (Fig. 1, Table 1).

### 4.1 Assay performance and key findings

Overall, the miniaturized precipitation assay captured the direction of the formulation effect for six out of seven compounds. In five cases, the magnitude of the formulation effect was also predicted reasonably well. The main findings can be grouped into formulation comparisons with relevant in vivo formulation effects including itraconazole, posaconazole, albendazole, and the comparison between the nanosized and micronized fenofibrate formulations. Of note, the magnitude of the albendazole effect was overestimated in vitro. Ritonavir, ziprasidone, and the comparison between the microcoated and micronized fenofibrate formulations showed no relevant formulation effect. Ibrutinib was the clearest case in which the assay indicated a formulation difference that was not apparent from the available in vivo data and therefore required more cautious quantitative interpretation.

For itraconazole, the precipitation assay reproduced both the direction and magnitude of the higher in vivo exposure observed for the oral solution compared to the capsule, although the corresponding in vitro AUC_abs_ difference was not statistically significant after adjustment (Fig. 1, Table 1). Both formulations can be considered enabling approaches, with the capsule based on an ASD and the oral solution on cyclodextrin complexation. The in vitro findings are consistent with the need for such enabling strategies, as concentrations of the neat active pharmaceutical ingredient (API) remained below the LOQ (data not shown), whereas both formulations yielded substantially higher concentrations (Fig. 2, Table 1). In the assay, the two formulations differed in their precipitation kinetics: the solution generated higher initial concentrations of dissolved drug, but precipitated upon transfer, whereas the fraction dissolved from the ASD capsule in SGFsp pH 2.0 remained stable during transfer. This illustrates that formulation performance may be enhanced through different mechanisms, including high initial solubilization as well as maintenance of dissolved concentrations after transfer. A methodological limitation for itraconazole was that the commercial itraconazole solution was not available and therefore reconstituted in-house based on the original formulation patent (Janssen Pharmaceutica N.V. 2340 Beerse, 1995). Although the preparation matched the reported composition using comparable ingredients except for the flavors, minor deviations from the marketed product cannot be excluded. However, the dissolved drug concentration was verified after preparation and deviated by less than 10% from the theoretical value. Comparable trends have been reported using more complex in vitro systems. In tiny-TIM and BioFLUX, the solution also performed better than the capsule, with approximately 2-fold higher bioaccessibility and 4-fold higher flux, respectively (Borbás et al., 2019; López Mármol et al., 2022). The same formulations were also evaluated in the BioGIT system, although direct comparison to our system is limited because BioGIT primarily reports luminal concentrations rather than absorbed fractions (Kourentas et al., 2016b). In the BioGIT, early intestinal concentrations of the solution were considerably higher than in the present assay, whereas later concentrations were more comparable. Notably, the authors suggested that early concentrations of the solution were likely overestimated relative to in vivo data. For the capsule, concentrations were comparable between the BioGIT and the present assay. Overall, itraconazole exemplifies a case in which the miniaturized precipitation assay successfully captured both the qualitative and quantitative formulation difference observed in vivo and showed formulation trends consistent with those reported for more complex in vitro systems.

The assay showed similarly good predictive performance for posaconazole, for which the assay captured the higher exposure observed for the acidified suspension compared with the neutral suspension (Fig. 1, Table 1). Mechanistically, the acidified suspension already contained a higher dissolved fraction of posaconazole in the donor prior to the transfer. Following a short period of supersaturation, posaconazole rapidly precipitated to concentrations close to the drug's crystalline FaSSIF solubility reported by Mayer et al. (approximately 2.8 μg/mL) (Mayer et al., 2025b). By contrast, the neutral suspension did not generate meaningful supersaturation and remained at or below the crystalline FaSSIF solubility (Mayer et al., 2025b). Comparable supersaturation and precipitation behavior has been reported previously (Hens et al., 2018). Of note, previous work indicated that inclusion of a permeation compartment did not substantially improve predictive performance for the investigated posaconazole formulations (Berben et al., 2018). Overall, posaconazole exemplifies successful qualitative and quantitative prediction of formulation differences using the miniaturized precipitation assay.

For ritonavir, the assay correctly reflected the reported similarity of the marketed tablet and the powder for oral suspension under fasted conditions, as shown in Fig. 1 and Table 1 (Xu et al., 2017). Mechanistically, both formulations are based on a polyvinylpyrrolidone-vinyl acetate (PVP-VA) ASD, but the powder for suspension has a smaller particle size (Xu et al., 2017), which likely explains the higher dissolved fraction in the donor prior to transfer compared with both the intact and ground tablet (Fig. 2). However, both formulations reached similar acceptor concentrations close to the amorphous FaSSIF solubility (56 μg/mL) reported by Indulkar et al. (Indulkar et al., 2018) and well above the crystalline solubility (11.5 μg/mL) reported by Fiolka et al. (Fiolka et al., 2020). This suggests that differences in donor dissolution had only limited impact once concentrations around the amorphous solubility were reached. The lower initial concentrations observed for the intact tablet were likely due to incomplete disintegration before transfer, as the ground tablet behaved similarly to the powder. Overall, the ritonavir formulations represent a case of good qualitative and quantitative agreement between the in vitro and the in vivo behavior, in which the assay correctly predicted the lack of a meaningful formulation effect.

Ziprasidone also showed good agreement between the assay outcome and the in vivo data, as both indicated only minor formulation effects (Fig. 1, Table 1). Notably, the concentration profiles suggested that continued dissolution rather than precipitation governed the observed behavior for both formulations, although overlapping dissolution and precipitation processes cannot be excluded. The measured acceptor concentrations were in the range of, or slightly above, the FaSSIF solubility of ziprasidone (4 μg/mL) reported by Riedmaier et al. (Riedmaier et al., 2020). The absence of pronounced precipitation is noteworthy given the reported tendency of ziprasidone salts to precipitate as the free base at intestinal pH values (Thombre et al., 2015). At the same time, the drug's precipitation behavior has been shown to strongly depend on the experimental conditions, with rapid dissolution without precipitation at pH 4, but rapid precipitation of the free base at pH 7.4 (Thombre et al., 2012b). In the present study, a reasonable explanation for the lack of precipitation may be the concentration of dissolved drug during transfer, which was likely too low to trigger nucleation under the tested conditions. Instead, the sustained dissolution was likely driven by solubilization in colloidal structures present in biorelevant medium, which is consistent with a high micelle-to-aqueous partition coefficient of ziprasidone (Thombre et al., 2012a). Thus, despite the absence of precipitation under the tested conditions, the assay predicted only a minor formulation effect, in line with the in vivo findings.

For albendazole, the assay correctly predicted the direction of the formulation effect, as the HP-β-CD formulation showed a substantially higher exposure than the marketed Eskazole® tablet both in vitro and in vivo (Rigter et al., 2004) (Fig. 1, Table 1). However, the magnitude of the effect was substantially overestimated in vitro. This overestimation may reflect a combination of assay-specific limitations and clinical confounders. From an in vitro perspective, the measurement of apparently dissolved drug may overestimate the fraction available for absorption, which may be particularly relevant for the cyclodextrin-based solution. From an in vivo perspective, the clinical study reported gastrointestinal side effects such as diarrhea at high cyclodextrin load (Rigter et al., 2004). Therefore, the “true” effect size in vivo remains unknown and may exceed the approximately 10-fold increase in bioavailability. In the precipitation assay, both formulations produced concentrations above the FaSSIF solubility of albendazole (approximately 1.4 μg/mL) reported by Mayer et al. (Mayer et al., 2025b), indicating that concentrations were not determined by crystalline solubility alone, but by the extent to which the drug remained solubilized throughout the experiment. The additional control experiments using a higher buffer concentration and hydrochloric acid indicated that acceptor acidification was not the primary driver of the superior performance of the cyclodextrin formulation. In contrast to the clinical study protocol (Rigter et al., 2004), the cyclodextrin formulation formed a suspension rather than a solution, with approximately 70–80% of the compound dissolved. The reason for this difference remains unclear, but is broadly consistent with previous work showing that albendazole solubilization in HP-β-CD depends strongly on preparation conditions (Pillai et al., 2017). From an experimental perspective, complete dissolution before transfer might have further increased the in vitro advantage over the tablet. However, a higher fraction of dissolved drug may have also increased supersaturation upon pH shift and thereby the risk of precipitation. Overall, this observation is not expected to change the qualitative interpretation of the albendazole results. In summary, the assay qualitatively predicted the formulation differences of albendazole but overestimated its quantitative effect.

For ibrutinib, there was a considerable discrepancy between the in vitro assay outcome and the formulation effect observed in vivo. Although the cyclodextrin-based solution and the marketed tablet are both absorbed almost completely in humans (FDA, Center for Drug Evaluation and Research, 2018; Scheers et al., 2015), the assay indicated a pronounced formulation effect in favor of the solution (Fig. 1, Table 1). One possible explanation is that the assay does not distinguish between molecularly and apparently dissolved drug, which may be particularly relevant for the cyclodextrin-based solution. In addition, the absence of an absorptive sink may have contributed to an underestimation of tablet performance. The assay-related limitations are discussed in more detail below. Taken alone, the in vitro AUC ratio would suggest a relevant formulation difference. However, the available in vivo data indicate that both formulations provide sufficient drug absorption despite their different in vitro performance.

Beyond the weakly basic compounds, fenofibrate provided an additional case to evaluate whether the assay can support formulation ranking of a poorly soluble neutral compound. The assay reproduced the reported fasted state in vivo rank order across three marketed formulations (nanosized tablet, 145 mg > microcoated tablet, 160 mg > micronized capsule, 200 mg) (Fig. 1, Table 1). The differences in relative bioavailability between these formulations are mainly driven by the underlying formulation technology and the drug's particle size. The 200 mg capsule contains micronized fenofibrate with particle sizes of approximately 5–15 μm, whereas in the 160 mg tablet, the micronized drug additionally underwent a microcoating process to facilitate the initial disintegration step (Guichard et al., 2000; Juenemann et al., 2011). In the 145 mg tablet, particle size was further reduced to approximately 500 nm (Juenemann et al., 2011). The trend towards higher exposure with smaller particle size was also reflected in the miniaturized precipitation assay: while dissolution in SGFsp pH 2.0 was incomplete for all formulations, the nanosized tablet showed the highest dissolved fraction before transfer. Overall acceptor concentrations remained low relative to the administered dose (AUC_rel_dose_ < 10%), in line with the generally poor aqueous solubility of fenofibrate (Hens et al., 2015; Juenemann et al., 2011). However, concentrations exceeded the FaSSIF solubility of 13.7 μg/mL reported by Vogt et al. for all three formulations during the experiment (Vogt et al., 2008). AUC_rel_dissolved_ values above 100% indicate that dissolution continued after transfer. Thus, assay outcomes appeared to be driven mainly by dissolution and solubilization kinetics rather than precipitation, suggesting that colloidal structures in FaSSIF provided additional solubilization capacity. Previously, Hens et al. compared the dissolution performance of the 145 mg and 200 mg fenofibrate tablets using a two-stage in vitro dissolution setup (Hens et al., 2015). Although Hens et al. indicated that a good correlation between the in vitro and in vivo data was only obtained using an additional permeation compartment, our small-scale precipitation assay differentiated between the formulations without an explicit permeation sink. These discrepancies may largely stem from methodological differences, particularly in terms of the applied volumes and the dose-to-volume ratio, as well as in the gastric dissolution setup and transfer conditions. Overall, fenofibrate shows that the assay captured the in vivo rank order qualitatively not only for the weak bases, but also for the neutral compound evaluated herein.

Taken together, the compound examples illustrate that formulation-dependent differences in the assay were not governed by precipitation alone. Depending on the compound and formulation, assay performance reflected differences in donor dissolution, initial solubilization, maintenance of supersaturation, precipitation kinetics, or continued dissolution after transfer. This is in line with the mechanistic consideration discussed by Price et al. for precipitation inhibition in supersaturating formulations, including increased apparent solubility, stabilization of supersaturation, and inhibition of nucleation or crystal growth (Price et al., 2019). Because the present assay captures the net apparent dissolved drug concentration over time, it does not distinguish between these individual mechanisms or between molecularly dissolved and solubilized drug.

To support interpretation of the in vitro AUC_rel_ values and to provide a semi-quantitative estimate of expected absorption, the absorption decision tree proposed earlier by Mayer et al. was applied on an exploratory basis to the formulations of the six weak bases (Mayer et al., 2025a). The decision tree combines AUC_rel_dose_ from the miniaturized precipitation assay with compound-specific effective permeability (P_eff_) values to assign weakly basic compounds to low, moderate, or high absorption categories. According to this approach, formulations with AUC_rel_dose_ values below 0.19 are assigned to the low absorption category, corresponding to a predicted fraction absorbed (f_a_) below 35%. For AUC_rel_dose_ values above 0.19, the compound's P_eff_ value is used to distinguish between moderate absorption, corresponding to a predicted f_a_ between 35% and 75%, and high absorption, corresponding to a predicted f_a_ above 75%. The P_eff_ cutoff separating these two categories is 1.8 * 10^−4^ cm/s (Mayer et al., 2025a). Importantly, the AUC_rel_dose_ threshold of 0.19 was not defined as an absolute or final cutoff in the previous work; therefore, compounds or formulations close to this threshold should be interpreted with caution. Since the decision tree has so far only been applied to unformulated drug substances, its application to formulations, as presented herein, should be regarded as exploratory. The decision tree was not applied to fenofibrate, as it was developed for weakly basic compounds.

Among the weak bases, ibrutinib was the most informative case for the decision-tree-based interpretation. Based on AUC_rel_dose_ alone, the tablet performed clearly worse than the cyclodextrin solution. However, the AUC_rel_dose_ value of the tablet was close to the cutoff of 0.19 used in the decision tree. Given its high permeability (FDA, Center for Drug Evaluation and Research, 2017), the tablet may therefore also be interpreted as being close to the high absorption category, whereas the cyclodextrin solution was clearly assigned to this category. In this absorption category, pronounced formulation-related differences in systemic exposure are not expected, as absorption is already high and the potential for further improvement by formulation approaches is limited. This may explain why the pronounced in vitro difference did not translate into a clinically meaningful formulation effect in vivo. Thus, the ibrutinib example illustrates that large numerical differences in AUC_rel_dose_ do not necessarily translate into relevant systemic exposure differences when absorption is expected to be high anyway. In such cases, the absorption category-based interpretation may be more informative than the magnitude of the in vitro AUC ratio alone. This emphasizes the importance of evaluating in vitro assay results together with further biopharmaceutical information such as permeability. However, this conclusion should be interpreted with caution, as ibrutinib was the only compound in the present dataset that showed high absorption in vivo while also exhibiting a pronounced formulation difference based on the in vitro AUC_rel_ values. A larger dataset would be needed to better define both assay-specific thresholds and the boundaries between absorption categories. However, the number of cases with reported fraction absorbed data *and* available high-quality comparative formulation studies is very limited.

For albendazole, the decision tree (Mayer et al., 2025a) supported the qualitative interpretation of the assay results. The marketed tablet was assigned to the low absorption category, whereas the cyclodextrin formulation was assigned to the high absorption category (Mayer et al., 2025b). These classifications are qualitatively consistent with the generally poor absorption of albendazole (approximately 5%) (Mayer et al., 2025b) and with the pronounced exposure enhancement observed in vivo. However, because the absorption value for this specific tablet may differ from the reported approximate value of 5%, the absolute absorption of the cyclodextrin formulation cannot be inferred from the in vivo fold-change alone. For itraconazole, posaconazole, and ziprasidone, the decision tree assigned all tested formulations to the low absorption category (Mayer et al., 2025a). This classification appears plausible given the solubility-limited behavior of these compounds and the low AUC_rel_dose_ values observed in the assay. For itraconazole, this is consistent with the known challenges in achieving sufficient intestinal absorption due to its low solubility and poor dissolution (Brouwers et al., 2017). For posaconazole and ziprasidone, human fraction absorbed data were also not available to directly validate the predicted category. However, the agreement between the in vitro and in vivo formulation comparisons shows that relevant relative formulation effects may still occur within the low absorption category and that the decision tree should therefore be used to contextualize, rather than replace, formulation-specific AUC comparisons. For ritonavir, the decision-tree classification depended on the assumed permeability, as ritonavir has been reported to be a borderline high-permeability compound (Bocci et al., 2022). Accordingly, both formulations were assigned either to the moderate or high absorption category (Mayer et al., 2025a). This is consistent with the similar in vivo performance of the two ASD-based formulations and with previous biphasic dissolution data predicting a fraction absorbed of approximately 65% (Xu et al., 2017).

Overall, these observations support the use of the decision tree as a complementary tool for interpreting assay outcomes, while its application to formulations remains exploratory.

### 4.2 Assay limitations and scope of applicability

One major hurdle we encountered was the identification of a suitable compound and formulation dataset for evaluating the miniaturized precipitation assay's ability to discriminate between formulations. Compound selection focused on poorly soluble weak bases with a potential risk of intestinal precipitation. The availability of human fasted state pharmacokinetic data for at least two formulations was another prerequisite. These two criteria substantially restricted the available dataset, as only one commercial formulation is available for most of the approved drugs, while early clinical studies often rely on prototype formulations for which formulation details are typically not publicly available. As a consequence of these restrictions, our final dataset comprised only six weakly basic drugs for which PK data for (at least) two formulations are available. The six weak bases were complemented by fenofibrate, a neutral drug, for which three formulations are commercially available. Thus, the present dataset was suitable for an initial evaluation of formulation comparisons but does not cover the full range of enabling formulation approaches used for poorly soluble drugs. Of note, formulations containing acidic polymers such as hydroxypropyl methylcellulose acetate succinate (HPMCAS) were not included, as these polymers often show a more complex, pH- and buffer-species-dependent dissolution and precipitation behavior (Jede et al., 2019b; Sakamoto and Sugano, 2021). To limit the degree of complexity, such polymers were outside the scope of the present work.

One widely discussed aspect of precipitation assays is the use (or omission) of sink conditions to mimic intestinal absorption (Bevernage et al., 2013; Hate et al., 2017; O'Dwyer et al., 2019; Pestieau and Evrard, 2017). To maintain simplicity and enable its use as a screening tool, the miniaturized assay applied in this study was designed without an absorptive sink. As a result, dissolved drug may accumulate in the acceptor compartment to concentrations that are unlikely to be maintained in vivo, where luminal drug is continuously removed by absorption. This may favor precipitation and reduce the likelihood of re-dissolution of precipitated drug, particularly for highly permeable compounds. Within the present dataset, such effects may be most relevant for the ibrutinib tablet and possibly also for the albendazole tablets, whereas for the other compounds there was no clear indication that the absence of an absorptive sink substantially limited interpretation. Although sink-related effects cannot be ruled out, the overall qualitative agreement with the available in vivo reference data suggests that the simplified setup was sufficient for screening the formulations evaluated in this study. Nevertheless, additional data are required to confirm this conclusion across a broader range of compounds and formulations.

A further important consideration is that the assay quantifies the apparently dissolved drug fraction and does not distinguish between the molecularly dissolved fraction and drug solubilized via complexation or micellar incorporation, for example in cyclodextrin complexes or bile salt micelles. This distinction is important because only the free, molecularly dissolved fraction is directly available for passive transcellular uptake. Accordingly, apparent dissolved concentrations may overestimate the fraction effectively available for absorption when a substantial proportion of drug is bound in complexes or intestinal micelles (Beig et al., 2013; Cuoco et al., 2023; Holm et al., 2016; Stappaerts and Augustijns, 2016; Westerberg and Wiklund, 2005). Moreover, in biorelevant media, competitive interactions between cyclodextrins and bile salts can shift the partitioning between free and solubilized drug in a concentration-dependent manner, thereby affecting supersaturation and precipitation behavior (Beig et al., 2013; Stappaerts and Augustijns, 2016). Because this solubility–permeability interplay is dynamic, its net impact on absorption is difficult to predict a priori and formulation design should therefore balance apparent solubility gains against potential permeability losses (Miller et al., 2011). In the present dataset, two out of three cyclodextrin formulations showed trends broadly consistent with in vivo data (itraconazole and albendazole), although the magnitude of the albendazole in vitro effect may be overestimated due to complexation and/or limitations of the in vivo reference study (Rigter et al., 2004). For ibrutinib, the observed difference between formulations could plausibly arise from considering only apparent dissolved concentrations for the cyclodextrin solution and/or sink-related effects for the tablet, as discussed above. In addition, this pronounced in vitro difference may not necessarily translate into a relevant in vivo difference, because even the tablet formulation with the lower AUC_rel_dose_ was close to the high absorption category when permeability was considered. Therefore, when a more quantitative interpretation is required for cyclodextrin-based formulations, permeability follow-up measurements (or assay variants including a permeation sink) should be considered.

A final consideration is that compound- or formulation-specific adaptations were applied in selected cases in the present study. Such adaptations are not intended to represent the standard use of the assay in early formulation screening but were required in some cases for experimental or product-related reasons. Some adaptations may also be relevant in future applications if they are needed to keep the assay conditions within the intended range. For example, highly acidic or buffered formulations, such as the albendazole and posaconazole formulations tested here, may shift the pH beyond the intended range. In such cases, pH control or adjustment may be appropriate to maintain conditions that are representative of the intended intestinal environment. By contrast, other adaptations were specific to the evaluation of the (marketed) drug products in this study and are less likely to be relevant for early prototype screening. Incomplete disintegration of the intact ritonavir tablet, for example, was related to the use of a marketed tablet and does not represent a general limitation of the miniaturized assay, as early prototypes would typically be tested directly at small scale. Similarly, the itraconazole pellets were removed from the capsule because previous work had shown comparable dissolution behavior and allowed the formulation to be tested in a manner more compatible with the miniaturized setup. Volume-related adaptations for albendazole were applied because the clinical study conditions were already known; such detailed information would usually not be available during early formulation screening. Overall, adaptations should be limited to cases in which they are needed to maintain meaningful assay conditions.

### 4.3 Practical implications for formulation screening

Based on the results of the present study, two earlier studies (Mayer et al., 2025b; Mayer et al., 2025a) and practical experience with the assay, we propose the following practical recommendations for the use of the miniaturized precipitation assay to guide compound selection (Mayer et al., 2025b; Mayer et al., 2025a) and formulation development (this study). The assay is thought to support both an early developability assessment at the drug substance level and at the preformulation level. In all cases, the assay is thought to complement other methods, such as PBBM and potentially also animal studies.

1) During drug discovery, the miniaturized precipitation assay can be performed with the drug substance to support compound ranking and selection. Combined with the absorption decision tree proposed earlier, formulation and biopharmaceutics scientists obtain a semi-quantitative estimate of the absorption properties of the compound(s) of interest (absorption categories: low, moderate, high absorption) (Mayer et al., 2025b; Mayer et al., 2025a).
2) Once a compound has been selected, the miniaturized precipitation assay can be used to compare the in vitro performance across various formulation prototypes. For example, if a drug is predicted to fall into the moderate or low absorption category as per (1) above, then the precipitation assay can provide insights into the performance of the prototype formulations in vivo and thus support formulation selection. By contrast, if the drug is predicted to fall into the high absorption category as per (1) above, then the application of the assay should be considered on a case-by-case basis, e.g., depending on the degree of drug and product knowledge and the level of uncertainty around them. In such cases, pronounced in vitro formulation differences may not necessarily translate into clinically meaningful exposure differences (see ibrutinib case example). The standard setup should be used whenever possible. Adaptations should only be considered if formulation- or compound-specific properties would otherwise compromise the intended assay conditions or the interpretability of the results. Examples include formulation-induced pH shifts beyond the intended range, poor formulation homogeneity, or administration conditions that are essential for the intended clinical use.
3) During clinical stage, further testing can be conducted in larger or more complex experimental systems to obtain more detailed mechanistic insights.

The guidance presented herein is based on a limited formulation dataset comprising 15 formulations of six weakly basic compounds and one neutral compound. As discussed in Section 4.2, identifying a suitable dataset for this type of assessment is challenging. The underlying absorption decision tree and its proposed thresholds were originally derived from a dataset of 17 basic drug substances and were applied on an exploratory basis to drug product formulations. Therefore, additional data are needed to confirm the validity of this approach and, if necessary, to refine the proposed thresholds and recommendations. Although several formulation approaches were investigated, including ASDs, cyclodextrin solutions, formulations differing in pH, and particle-size-reduced formulations, many other formulation strategies remain to be evaluated, and the guidance may need to be adapted accordingly.

## 5 Conclusion

Tested across six weakly basic compounds and one neutral compound, the results from the miniaturized precipitation assay showed good qualitative agreement with fasted state human PK data for capturing formulation-dependent effects. The assay therefore appears to be well suited as an efficient, small-scale screening tool particularly for early formulation development. Key limitations might include the absence of an absorptive sink and the lack of quantification of the molecularly dissolved drug fraction, which can complicate quantitative interpretation for solubilizing/complexing systems (e.g., cyclodextrin-containing formulations). Although this was not evident in the present compound/formulation dataset, in vitro assays including a permeation compartment may provide additional mechanistic insight for such formulations when needed. Finally, combining assay results with the absorption decision tree published earlier may further support formulation ranking (Mayer et al., 2025a). However, given the comparably small dataset used herein, further validation across a broader range of compounds and formulations is needed.

## CRediT authorship contribution statement

**Sara Mayer:** Writing – review & editing, Writing – original draft, Visualization, Project administration, Methodology, Investigation, Formal analysis, Conceptualization. **Katharina Krollik:** Writing – review & editing, Methodology, Conceptualization. **Christian Wagner:** Writing – review & editing, Methodology, Conceptualization. **Andreas Lehmann:** Writing – review & editing, Methodology, Conceptualization. **Jacob Spanheimer:** Writing – review & editing, Methodology, Investigation, Formal analysis. **Werner Weitschies:** Writing – review & editing, Supervision, Methodology, Conceptualization.

## Declaration of generative AI and AI-assisted technologies in the writing process

During the preparation of this work, the authors used GPT 5.4 to check spelling and grammar and improve readability. After using this tool/service, the authors reviewed and edited the content as needed and take full responsibility for the content of the publication.

## Funding

This research did not receive any specific grant from funding agencies in the public, commercial, or not-for-profit sectors.

## Declaration of competing interest

The authors declare the following financial interests/personal relationships which may be considered as potential competing interests: Sara Mayer reports financial support was provided by Merck KGaA. Katharina Krollik reports financial support was provided by Merck KGaA. Christian Wagner reports financial support was provided by Merck KGaA. Andreas Lehmann reports financial support was provided by Merck KGaA. Jacob Spanheimer reports financial support was provided by Merck KGaA. Sara Mayer reports a relationship with Merck KGaA that includes: employment and equity or stocks. Katharina Krollik reports a relationship with Merck KGaA that includes: employment and equity or stocks. Christian Wagner reports a relationship with Merck KGaA that includes: employment and equity or stocks. Andreas Lehmann reports a relationship with Merck KGaA that includes: employment and equity or stocks. Jacob Spanheimer reports a relationship with Merck KGaA that includes: employment and equity or stocks. If there are other authors, they declare that they have no known competing financial interests or personal relationships that could have appeared to influence the work reported in this paper.

## Data Availability

Data will be made available on request.

## References

[bb0005] Andreas C.J., Rosenberger J., Butler J., Augustijns P., McAllister M., Abrahamsson B., Dressman J. (2018). Introduction to the OrBiTo decision tree to select the most appropriate in vitro methodology for release testing of solid oral dosage forms during development. Eur. J. Pharm. Biopharm..

[bb0010] Augustijns P., Wuyts B., Hens B., Annaert P., Butler J., Brouwers J. (2014). A review of drug solubility in human intestinal fluids: Implications for the prediction of oral absorption. Eur. J. Pharm. Sci..

[bb0015] Barker R., Abrahamsson B., Kruusmägi M. (2014). Application and validation of an advanced gastrointestinal in vitro model for the evaluation of drug product performance in pharmaceutical development. J. Pharm. Sci..

[bb0020] Beig A., Agbaria R., Dahan A. (2013). Oral delivery of lipophilic drugs: the tradeoff between solubility increase and permeability decrease when using cyclodextrin-based formulations. PLoS One.

[bb0025] Benjamini Y., Hochberg Y. (1995). Controlling the false discovery rate: a practical and powerful approach to multiple testing. J. R. Stat. Soc. B. Methodol..

[bb0030] Berben P., Brouwers J., Augustijns P. (2018). The artificial membrane insert system as predictive tool for formulation performance evaluation. Int. J. Pharm..

[bb0035] Bevernage J., Brouwers J., Brewster M.E., Augustijns P. (2013). Evaluation of gastrointestinal drug supersaturation and precipitation: strategies and issues. Int. J. Pharm..

[bb0040] Bocci G., Oprea T.I., Benet L.Z. (2022). State of the art and uses for the Biopharmaceutics Drug Disposition Classification System (BDDCS): new additions, revisions, and citation references. AAPS J..

[bb0045] Borbás E., Kádár S., Tsinman K., Tsinman O., Csicsák D., Takács-Novák K., Völgyi G., Sinkó B., Pataki H. (2019). Prediction of bioequivalence and food effect using flux- and solubility-based methods. Mol. Pharm..

[bb0050] Boyd B.J., Bergström C.A.S., Vinarov Z., Kuentz M., Brouwers J., Augustijns P., Brandl M., Bernkop-Schnürch A., Shrestha N., Préat V., Müllertz A., Bauer-Brandl A., Jannin V. (2019). Successful oral delivery of poorly water-soluble drugs both depends on the intraluminal behavior of drugs and of appropriate advanced drug delivery systems. Eur. J. Pharm. Sci..

[bb0055] Brouwers J., Brewster M.E., Augustijns P. (2009). Supersaturating drug delivery systems: the answer to solubility-limited oral bioavailability?. J. Pharm. Sci..

[bb0060] Brouwers J., Geboers S., Mols R., Tack J., Augustijns P. (2017). Gastrointestinal behavior of itraconazole in humans - part 1: supersaturation from a solid dispersion and a cyclodextrin-based solution. Int. J. Pharm..

[bb0065] Cuoco A., Eriksen J.B., Luppi B., Brandl M., Bauer-Brandl A. (2023). When interactions between bile salts and cyclodextrin cause a negative food effect: dynamic dissolution/permeation studies with Itraconazole (Sporanox®) and biomimetic media. J. Pharm. Sci..

[bb0070] FDA, Center for Drug Evaluation and Research (2001). Tricor® Fenofibrate Tablets: Clinical Pharmacology and Biopharmaceutics Review(s) for NDA 21203. https://www.accessdata.fda.gov/drugsatfda_docs/nda/2001/21-203_Tricor_biopharmr.pdf.

[bb0075] FDA, Center for Drug Evaluation and Research (2005). Ziprasidone Oral Suspension, 10 mg/mL: Clinical Pharmacology and Biopharmaceutics Review(s) for NDA 21483. https://www.accessdata.fda.gov/drugsatfda_docs/nda/2006/021483s000_ClinPharmR.pdf.

[bb0080] FDA, Center for Drug Evaluation and Research (2017). Imbruvica® (Ibrutinib) Tablets: Product Quality Review(s) for NDA 210563. https://www.accessdata.fda.gov/drugsatfda_docs/nda/2018/210563Orig1s000,210563Orig2s000ChemR.pdf.

[bb0085] FDA, Center for Drug Evaluation and Research (2018). Imbruvica® (Ibrutinib) Tablets: Clinical Pharmacology and biopharmaceutics review(s) for NDA 210563. https://www.accessdata.fda.gov/drugsatfda_docs/nda/2018/210563Orig1s000,210563Orig2s000ClinPharmR.pdf.

[bb0090] Fiolka T., Dressman J.B. (2018). Development, current applications and future roles of biorelevant two-stage in vitro testing in drug development. J. Pharm. Pharmacol..

[bb0095] Fiolka T., van den Abeele J., Augustijns P., Arora S., Dressman Jennifer (2020). Biorelevant two-stage in vitro testing for rDCS classification and in PBPK modeling-case example ritonavir. J. Pharm. Sci..

[bb0100] Galia E., Nicolaides E., Hörter D., Löbenberg R., Reppas C., Dressman J.B. (1998). Evaluation of various dissolution media for predicting in vivo performance of class I and II drugs. Pharm. Res..

[bb0105] Gastwirth J.L., Gel Y.R., Miao W. (2009). The impact of Levene’s test of equality of variances on statistical theory and practice. Stat. Sci..

[bb0110] Guichard J.P., Blouquin P., Qing Y. (2000). A new formulation of fenofibrate: suprabioavailable tablets. Curr. Med. Res. Opin..

[bb0115] Hate S.S., Reutzel-Edens S.M., Taylor L.S. (2017). Absorptive dissolution testing of supersaturating systems: impact of absorptive sink conditions on solution phase behavior and mass transport. Mol. Pharm..

[bb0120] Hens B., Brouwers J., Corsetti M., Augustijns P. (2015). Gastrointestinal behavior of nano- and microsized fenofibrate: in vivo evaluation in man and in vitro simulation by assessment of the permeation potential. Eur. J. Pharm. Sci..

[bb0125] Hens B., Brouwers J., Corsetti M., Augustijns P. (2016). Supersaturation and precipitation of posaconazole upon entry in the upper small intestine in humans. J. Pharm. Sci..

[bb0130] Hens B., Bermejo M., Tsume Y., Gonzalez-Alvarez I., Ruan H., Matsui K., Amidon G.E., Cavanagh K.L., Kuminek G., Benninghoff G., Fan J., Rodríguez-Hornedo N., Amidon G.L. (2018). Evaluation and optimized selection of supersaturating drug delivery systems of posaconazole (BCS class 2b) in the gastrointestinal simulator (GIS): an in vitro-in silico-in vivo approach. Eur. J. Pharm. Sci..

[bb0135] Holm R., Olesen N.E., Hartvig R.A., Jørgensen E.B., Larsen D.B., Westh P. (2016). Effect of cyclodextrin concentration on the oral bioavailability of danazol and cinnarizine in rats. Eur. J. Pharm. Biopharm..

[bb0140] Indulkar A.S., Gao Y., Raina S.A., Zhang G.G.Z., Taylor L.S. (2018). Crystallization from supersaturated solutions: role of lecithin and composite simulated intestinal fluid. Pharm. Res..

[bb0145] Janssen Pharmaceutica N.V. 2340 Beerse (1995). https://patentscope.wipo.int/search/docs2/pct/WO1995008993/pdf/5VAr0z2-99OOWQlGfL4jIF0RBAhRFCf2x7TVQdGP0RvnXF5noAeIB_Zp8OVJC3V.

[bb0150] Jede C., Wagner C., Kubas H., Weber C., Weigandt M., Koziolek M., Weitschies W. (2019). Automated small-scale in vitro transfer model as screening tool for the prediction of in vivo-dissolution and precipitation of poorly solubles. Int. J. Pharm..

[bb0155] Jede C., Wagner C., Kubas H., Weigandt M., Weber C., Lecomte M., Badolo L., Koziolek M., Weitschies W. (2019). Improved prediction of in vivo supersaturation and precipitation of poorly soluble weakly basic drugs using a biorelevant bicarbonate buffer in a gastrointestinal transfer model. Mol. Pharm..

[bb0160] Juenemann D., Jantratid E., Wagner C., Reppas C., Vertzoni M., Dressman J.B. (2011). Biorelevant in vitro dissolution testing of products containing micronized or nanosized fenofibrate with a view to predicting plasma profiles. Eur. J. Pharm. Biopharm..

[bb0165] Klein S. (2010). The use of biorelevant dissolution media to forecast the in vivo performance of a drug. AAPS J..

[bb0170] Kostewicz E.S., Wunderlich M., Brauns U., Becker R., Bock T., Dressman J.B. (2004). Predicting the precipitation of poorly soluble weak bases upon entry in the small intestine. J. Pharm. Pharmacol..

[bb0175] Kou D., Zhang C., Yiu H., Ng T., Lubach J.W., Janson M., Mao C., Durk M., Chinn L., Winter H., Wigman L., Yehl P. (2018). In vitro, in silico, and in vivo assessments of intestinal precipitation and its impact on bioavailability of a BCS class 2 basic compound. Mol. Pharm..

[bb0180] Kourentas A., Vertzoni M., Stavrinoudakis N., Symillidis A., Brouwers J., Augustijns P., Reppas C., Symillides M. (2016). An in vitro biorelevant gastrointestinal transfer (BioGIT) system for forecasting concentrations in the fasted upper small intestine: Design, implementation, and evaluation. Eur. J. Pharm. Sci..

[bb0185] Kourentas A., Vertzoni M., Symillides M., Hens B., Brouwers J., Augustijns P., Reppas C. (2016). In vitro evaluation of the impact of gastrointestinal transfer on luminal performance of commercially available products of posaconazole and itraconazole using BioGIT. Int. J. Pharm..

[bb0190] López Mármol Á., Fischer P.L., Wahl A., Schwöbel D., Lenz V., Sauer K., Koziolek M. (2022). Application of tiny-TIM as a mechanistic tool to investigate the in vitro performance of different itraconazole formulations under physiologically relevant conditions. Eur. J. Pharm. Sci..

[bb0195] Mayer S., Krollik K., Wagner C., Wittmann J., Lehmann A. (2025). Precipitation as key parameter for early oral drug absorption predictions, Part II: development of a predictive model. Eur. J. Pharm. Sci..

[bb0200] Mayer S., Wagner C., Lehmann A., Fang Z., Weitschies W., Krollik K. (2025). Precipitation as key parameter for early oral drug absorption predictions, part I: acquisition of critical data. Eur. J. Pharm. Sci..

[bb0205] Miller J.M., Beig A., Krieg B.J., Carr R.A., Borchardt T.B., Amidon G.E., Amidon G.L., Dahan A. (2011). The solubility-permeability interplay: mechanistic modeling and predictive application of the impact of micellar solubilization on intestinal permeation. Mol. Pharm..

[bb0210] Mudie D.M., Murray K., Hoad C.L., Pritchard S.E., Garnett M.C., Amidon G.L., Gowland P.A., Spiller R.C., Amidon G.E., Marciani L. (2014). Quantification of gastrointestinal liquid volumes and distribution following a 240 mL dose of water in the fasted state. Mol. Pharm..

[bb0215] O'Dwyer P.J., Litou C., Box K.J., Dressman J.B., Kostewicz E.S., Kuentz M., Reppas C. (2019). In vitro methods to assess drug precipitation in the fasted small intestine - a PEARRL review. J. Pharm. Pharmacol..

[bb0220] Pestieau A., Evrard B. (2017). In vitro biphasic dissolution tests and their suitability for establishing in vitro-in vivo correlations: a historical review. Eur. J. Pharm. Sci..

[bb0225] Pillai K., Akhter J., Morris D.L. (2017). Super aqueous solubility of albendazole in β-cyclodextrin for parenteral application in cancer therapy. J. Cancer.

[bb0230] Price D.J., Ditzinger F., Koehl N.J., Jankovic S., Tsakiridou G., Nair A., Holm R., Kuentz M., Dressman J.B., Saal C. (2019). Approaches to increase mechanistic understanding and aid in the selection of precipitation inhibitors for supersaturating formulations – a PEARRL review. J. Pharm. Pharmacol..

[bb0235] Riedmaier A.E., DeMent K., Huckle J., Bransford P., Stillhart C., Lloyd R., Alluri R., Basu S., Chen Y., Dhamankar V., Dodd S., Kulkarni P., Olivares-Morales A., Peng C.-C., Pepin X., Ren X., Tran T., Tistaert C., Heimbach T., Kesisoglou F., Wagner C., Parrott N. (2020). Use of Physiologically Based Pharmacokinetic (PBPK) modeling for predicting drug-food interactions: an industry perspective. AAPS J..

[bb0240] Rigter I.M., Schipper H.G., Koopmans R.P., van Kan H.J.M., Frijlink H.W., Kager P.A., Guchelaar H.-J. (2004). Relative bioavailability of three newly developed albendazole formulations: a randomized crossover study with healthy volunteers. Antimicrob. Agents Chemother..

[bb0245] Rudolph N., Charbe N., Plano D., Shoyaib A.A., Pal A., Boyce H., Zhao L., Wu F., Polli J., Dressman Jennifer, Cristofoletti R. (2025). A physiologically based biopharmaceutics modeling (PBBM) framework for characterizing formulation-dependent food effects: Paving the road towards fed state virtual BE studies for itraconazole amorphous solid dispersions. Eur. J. Pharm. Sci..

[bb0250] Ruxton G.D. (2006). The unequal variance t-test is an underused alternative to Student's t-test and the Mann–Whitney U test. Behav. Ecol..

[bb0255] Sakamoto A., Sugano K. (2021). Dissolution kinetics of nifedipine-ionizable polymer amorphous solid dispersion: comparison between bicarbonate and phosphate buffers. Pharm. Res..

[bb0260] Scheers E., Leclercq L., de Jong J., Bode N., Bockx M., Laenen A., Cuyckens F., Skee D., Murphy J., Sukbuntherng J., Mannens G. (2015). Absorption, metabolism, and excretion of oral ^14^C radiolabeled ibrutinib: an open-label, phase I, single-dose study in healthy men. Drug Metab. Dispos..

[bb0265] Stappaerts J., Augustijns P. (2016). Displacement of itraconazole from cyclodextrin complexes in biorelevant media: in vitro evaluation of supersaturation and precipitation behavior. Int. J. Pharm..

[bb0270] Thombre A.G., Caldwell W.B., Friesen D.T., McCray S.B., Sutton S.C. (2012). Solid nanocrystalline dispersions of ziprasidone with enhanced bioavailability in the fasted state. Mol. Pharm..

[bb0275] Thombre A.G., Shah J.C., Sagawa K., Caldwell W.B. (2012). In vitro and in vivo characterization of amorphous, nanocrystalline, and crystalline ziprasidone formulations. Int. J. Pharm..

[bb0280] Thombre A.G., Shamblin S.L., Malhotra B.K., Connor A.L., Wilding I.R., Caldwell W.B. (2015). Pharmacoscintigraphy studies to assess the feasibility of a controlled release formulation of ziprasidone. J. Control. Release.

[bb0285] U.S. Food and Drug Administration. Drugs@FDA: FDA-Approved Drugs. (n.d.). https://www.accessdata.fda.gov/scripts/cder/daf/index.cfm.

[bb0290] Vogt M., Kunath K., Dressman J.B. (2008). Dissolution enhancement of fenofibrate by micronization, cogrinding and spray-drying: comparison with commercial preparations. Eur. J. Pharm. Biopharm..

[bb0295] Welch B.L. (1947). The generalization of ‘student’s’ problem when several different population variances are involved. Biometrika.

[bb0300] Westerberg G., Wiklund L. (2005). β-cyclodextrin reduces bioavailability of orally administered [3H]Benzo[a]pyrene in the rat. J. Pharm. Sci..

[bb0305] Xu H., Vela S., Shi Y., Marroum P., Gao P. (2017). In vitro characterization of ritonavir drug products and correlation to human in vivo performance. Mol. Pharm..

