# Supplementary material for: Applicability of a miniaturized precipitation assay as an early-stage screening tool for prototype formulation development
Source: Int J Pharm X. 2026 Oct 1;12:100677. doi: 10.1016/j.ijpx.2026.100677 (PMC13675896; doi:10.1016/j.ijpx.2026.100677)
Supplement: Supplementary file 1 [file mmc1.docx]

**Supplementary material: “Applicability of a miniaturized precipitation assay as an early-stage screening tool for prototype formulation development”**

Sara Mayer^a,b^, Katharina Krollik^b^, Christian Wagner^b^, Andreas Lehmann^b^, Jacob Spanheimer^b^, Werner Weitschies^a^*

^a^Institute of Pharmacy, Department of Biopharmaceutics and Pharmaceutical Technology, University of Greifswald, Felix-Hausdorff-Straße 3, 17489 Greifswald, Germany

^b^Global Drug Product Development, Merck KGaA, Frankfurter Straße 250, 64293 Darmstadt, Germany

*Corresponding author; werner.weitschies@uni-greifswald.de

**Analytical methods**

For ritonavir, an adjusted method was applied with a total run time of 7.5 min. The method consisted of an isocratic hold at 95 % mobile phase A (water with 0.1 % trifluoroacetic acid) for 1 minute, followed by a gradient to 95 % mobile phase B (acetonitrile) over 4 min. At 5.5 min, conditions returned to initial state.

For ziprasidone, an adjusted method was utilized which had a total run time of 10 min. It consisted of a gradient changing from 95 % mobile phase A (ammonium acetate buffer pH 8.0) to 47.5 % mobile phase B (acetonitrile) over 7 minutes, then to 95 % mobile phase B within 1 minute, followed by a return to initial conditions between 9 and 9.1 minutes of total run time.

The limit of quantification (LOQ) was determined using the same Ultra-High Performance Liquid Chromatography (UHPLC) method used for quantification of the compounds, following the *International Council for Harmonisation (ICH) Guideline Q2(R2): Validation of analytical procedures* based on the standard deviation of the response and the slope of the calibration curve (International Council for Harmonisation, 2023). The calculated LOQs and wavelengths used for detection are summarized in Table S1.

Table S1: Detection wavelengths and LOQs of the investigated compounds.

| **Compound** | **Wavelength used for detection (nm)** | **LOQ (µg/mL)** |
| --- | --- | --- |
| Albendazole | 302 | 0.05 |
| Ibrutinib | 230 | 0.13 |
| Itraconazole | 258 | 0.32 |
| Posaconazole | 258 | 0.07 |
| Ritonavir | 240 | 0.23 |
| Ziprasidone hydrochloride monohydrate | 230 | 0.49 |
| Fenofibrate | 287 | 0.50 |

LOQ = limit of quantification.

**Physicochemical characteristics of investigated compounds**

Table S2: Physicochemical properties of the compounds investigated in this study.

| **Compound** | **MW (g/mol)** | **Basic pKa** | **Lipophilicity** | **BCS class** |
| --- | --- | --- | --- | --- |
| Albendazole | 265.3 | 3.6  (G. S. Paulekuhn et al., 2013) | 3.46  (Log*P*)  (Rivera et al., 2007) | II/IV  (Bocci et al., 2022) |
| Ibrutinib | 440.5 | 3.74  (FDA, Center for Drug Evaluation and Research, 2013) | 3.97  (Log*P*)  (FDA, Center for Drug Evaluation and Research, 2013) | II  (FDA, Center for Drug Evaluation and Research, 2017) |
| Itraconazole | 705.6 | 3.9^a^ | 7.31 (Log*D*_(pH 6.5)_) ^a^ | II  (Bocci et al., 2022) |
| Posaconazole | 700.8 | 4 ^a^ | 5.41 (Log*D*_(pH 6.5)_) ^a^ | II  (Hens et al., 2018) |
| Ritonavir | 720.9 | 2.6 ^a^ | 5.22 (Log*D*_(pH 6.5)_) ^a^ | II/IV  (Bocci et al., 2022) |
| Ziprasidone hydrochloride monohydrate | 467.4 | 6.58  (Sutton et al., 2017) | 4.1 (cLog*D*_(pH 7.4)_)  (Riedmaier et al., 2020) | II  (Riedmaier et al., 2020) |
| Fenofibrate | 360.8 | - | 5.28 (Log*D*_(pH 6.5)_) ^a^ | II  (Hens et al., 2015) |

MW = molecular weight, BCS = Biopharmaceutics Classification System, cLogD = calculated LogD.

^a^Predicted values from the literature (Augustijns et al., 2014)

**Additional data from in vitro precipitation assay**

Table S3: Extended summary of the investigated formulations and resulting AUC values based on Table 1 of the main manuscript.

| **Compound** | **Dose**  **(mg)** | **Formulation** | **AUC_abs_ (µg/mL*min)** | **In vitro AUC_rel_dose_ (%)** | **In vitro AUC_rel_dose_ (%)** |
| --- | --- | --- | --- | --- | --- |
| Albendazole | 800 | HP-ꞵ-CD solution | 363 *10^2^ ± 6 *10^2^ (condition 1)  3468 *10 ± 19 *10 (condition 2)  343 *10² ± 6*10²  (condition 3) | Condition 1: see main script  78.2 ± 0.5 (condition 2)  77.4 ± 1.3  (condition 3) | Condition 1: see main script  94.4 ± 0.6 (condition 2)  94.4 ± 1.6  (condition 3) |
|  |  | Eskazole® tablet | 64 *10 ± 10 *10 | - | - |
| Ibrutinib | 140 | HP-ꞵ-CD solution | 1015 *10 ± 18 *10 | - | - |
|  |  | Imbruvica® tablet | 186 *10 ± 6 *10 | - | - |

Table S3: Continued.

| Itraconazole | 200 | Sporanox® HP-ꞵ-CD solution | 16 *10^2^ ± 5 *10^2^ | - | - |
| --- | --- | --- | --- | --- | --- |
|  |  | Sporanox® capsule (ASD) | 49 *10 ± 5 *10 | - | - |
| Posaconazole | 40 | Noxafil® suspension (acidified) | 193 ± 14 | - | - |
|  |  | Noxafil® suspension (neutral) | 113.2 ± 1.5 | - | - |
| Ritonavir | 100 | Norvir® powder for suspension (ASD) | 224 *10 ± 6 *10 | - | - |
|  |  | Norvir® tablet (ASD) | 2161 ± 11  (ground)  174 *10 ± 14 *10 (intact) | Ground: see main script  23.3 ± 1.9 (intact) | Ground: see main script  90 ± 7  (intact) |
| Ziprasidone hydrochloride monohydrate | 20 (free base)/  22.64 (salt) | Zeldox® capsule | 262 ± 11 | - | - |
|  |  | Zeldox® suspension | 294 ± 13 | - | - |
| Fenofibrate | 145 | Lipidil 145 one® tablet (nanosized) | 98 *10 ± 5 *10 | - | - |
|  | 160 | Lipidil-Ter® tablet (micronized and microcoated) | 77 *10 ± 6 *10 | - | - |
|  | 200 | Lipidil® capsule (micronized) | 63 *10 ± 10 *10 | - | - |

The AUC values refer to a 45 min time interval and are given as mean ± SD, n = 3. For albendazole solution, condition 1 refers to the standard setup, condition 2 to the higher concentrated buffer, and condition 3 to hydrochloric acid as acidifier. HP-ꞵ-CD = Hydroxypropyl-beta-cyclodextrin, ASD = amorphous solid dispersion, AUC = area under the curve, AUC_rel_dose_ = relative area under the curve based on dose, AUC_abs_ = absolute area under the curve, SD = standard deviation.

Figure S1: Concentration profiles of additional conditions of the albendazole solution, given as mean ± SD, n = 3. Alb 2 refers to the condition with increased buffer concentration and Alb 3 refers to the condition in which citric acid was replaced by hydrochloric acid. For further details, see the main manuscript. Alb = albendazole, SD = standard deviation.

Figure 2: Concentration profile of the intact ritonavir tablet (Norvir®), given as mean ± SD, n = 3. For further details, see the main manuscript. SD = standard deviation.

References

Augustijns, P., Wuyts, B., Hens, B., Annaert, P., Butler, J., Brouwers, J., 2014. A review of drug solubility in human intestinal fluids: Implications for the prediction of oral absorption. Eur. J. Pharm. Sci. 57, 322–332. https://doi.org/10.1016/j.ejps.2013.08.027.

Bocci, G., Oprea, T.I., Benet, L.Z., 2022. State of the Art and Uses for the Biopharmaceutics Drug Disposition Classification System (BDDCS): New Additions, Revisions, and Citation References. AAPS J. 24, 37, 1–17. https://doi.org/10.1208/s12248-022-00687-0.

FDA, Center for Drug Evaluation and Research, 2013. Imbruvica™: Clinical Pharmacology and Biopharmaceutics Review(s) for NDA 205552. https://www.accessdata.fda.gov/drugsatfda_docs/nda/2013/205552orig1s000clinpharmr.pdf (accessed 16 September 2025).

FDA, Center for Drug Evaluation and Research, 2017. Imbruvica® (Ibrutinib) Tablets: Product Quality Review(s) for NDA 210563. https://www.accessdata.fda.gov/drugsatfda_docs/nda/2018/210563Orig1s000,210563Orig2s000ChemR.pdf (accessed 16 September 2025).

G. S. Paulekuhn, J. B. Dressman, C. Saal, 2013. Salt screening and characterization for poorly soluble, weak basic compounds: case study albendazole. Pharmazie 68, 555–564. https://doi.org/10.1691/ph.2013.6507.

Hens, B., Bermejo, M., Tsume, Y., Gonzalez-Alvarez, I., Ruan, H., Matsui, K., Amidon, G.E., Cavanagh, K.L., Kuminek, G., Benninghoff, G., Fan, J., Rodríguez-Hornedo, N., Amidon, G.L., 2018. Evaluation and optimized selection of supersaturating drug delivery systems of posaconazole (BCS class 2b) in the gastrointestinal simulator (GIS): An in vitro-in silico-in vivo approach. Eur. J. Pharm. Sci. 115, 258–269. https://doi.org/10.1016/j.ejps.2018.01.039.

Hens, B., Brouwers, J., Corsetti, M., Augustijns, P., 2015. Gastrointestinal behavior of nano- and microsized fenofibrate: In vivo evaluation in man and in vitro simulation by assessment of the permeation potential. Eur. J. Pharm. Sci. 77, 40–47. https://doi.org/10.1016/j.ejps.2015.05.023.

International Council for Harmonisation, 2023. ICH Q2(R2) Guideline on validation of analytical procedures. https://www.ema.europa.eu/en/ich-q2r2-validation-analytical-procedures-scientific-guideline (accessed 5 March 2026).

Riedmaier, A.E., DeMent, K., Huckle, J., Bransford, P., Stillhart, C., Lloyd, R., Alluri, R., Basu, S., Chen, Y., Dhamankar, V., Dodd, S., Kulkarni, P., Olivares-Morales, A., Peng, C.-C., Pepin, X., Ren, X., Tran, T., Tistaert, C., Heimbach, T., Kesisoglou, F., Wagner, C., Parrott, N., 2020. Use of Physiologically Based Pharmacokinetic (PBPK) Modeling for Predicting Drug-Food Interactions: an Industry Perspective. AAPS J. 22, 123. https://doi.org/10.1208/s12248-020-00508-2.

Rivera, J.C., Yépez-Mulia, L., Hernández-Campos, A., Moreno-Esparza, R., Castillo, R., Navarrete-Vázquez, G., Fuentes-Noriega, I., Jung-Cook, H., 2007. Biopharmaceutic evaluation of novel anthelmintic (1H-benzimidazol-5(6)-yl)carboxamide derivatives. Int. J. Pharm. 343, 159–165. https://doi.org/10.1016/j.ijpharm.2007.05.013.

Sutton, S.C., Nause, R., Gandelman, K., 2017. The impact of gastric pH, volume, and emptying on the food effect of ziprasidone oral absorption. AAPS J. 19, 1084–1090. https://doi.org/10.1208/s12248-017-0065-9.
